# Effect of Exercise on Chemotherapy-Induced Peripheral Neuropathy Among Patients Treated for Ovarian Cancer: A Secondary Analysis of a Randomized Clinical Trial

**DOI:** 10.1001/jamanetworkopen.2023.26463

**Published:** 2023-08-01

**Authors:** Anlan Cao, Brenda Cartmel, Fang-Yong Li, Linda T. Gottlieb, Maura Harrigan, Jennifer A. Ligibel, Radhika Gogoi, Peter E. Schwartz, Denise A. Esserman, Melinda L. Irwin, Leah M. Ferrucci

**Affiliations:** 1 Department of Chronic Disease Epidemiology, Yale School of Public Health, New Haven, Connecticut; 2 Yale Cancer Center, New Haven, Connecticut; 3 Dana-Farber Cancer Institute, Boston, Massachusetts; 4 Wayne State University, Detroit, Michigan; 5 Yale School of Medicine, New Haven, Connecticut; 6 Department of Biostatistics, Yale School of Public Health, New Haven, Connecticut

## Abstract

**Importance:**

Chemotherapy-induced peripheral neuropathy (CIPN), one of the most common and severe adverse effects of chemotherapy, is associated with worse quality of life among survivors of ovarian cancer. Currently, there is no effective treatment for CIPN.

**Objective:**

To evaluate the effect of a 6-month aerobic exercise intervention vs attention-control on CIPN among women treated for ovarian cancer in the Women’s Activity and Lifestyle Study in Connecticut (WALC) to provide evidence to inform the guidelines and recommendations for prevention or treatment of CIPN.

**Design, Setting, and Participants:**

This prespecified secondary analysis evaluated the Women’s Activity and Lifestyle Study in Connecticut (WALC), a multicentered, open-label, population-based, phase 3 randomized clinical trial of an aerobic exercise intervention vs attention control for CIPN in patients who were diagnosed with ovarian cancer. Only WALC participants who received chemotherapy were included in this analysis. Participants were randomized 1:1 to either a 6-month aerobic exercise intervention or to attention control. All analyses were conducted between September 2022 and January 2023.

**Interventions:**

The exercise intervention consisted of home-based moderate-intensity aerobic exercise facilitated by weekly telephone counseling from an American College of Sports Medicine/American Cancer Society–certified cancer exercise trainer. Attention control involved weekly health education telephone calls from a WALC staff member.

**Main Outcomes and Measure:**

Change in CIPN was the primary outcome in this secondary analysis. This outcome was represented by CIPN severity, which was self-measured by participants at baseline and 6 months using the Functional Assessment of Cancer Therapy/Gynecologic Oncology Group–Neurotoxicity scale, with a score range of 0 to 44. A mixed-effects model was used to assess the 6-month change in CIPN between the exercise intervention and attention control arms.

**Results:**

Of the 134 participants (all females; mean [SD] age, 57.5 [8.3] years) included in the analysis, 69 were in the exercise intervention arm and 65 were in the attention control arm. The mean (SD) time since diagnosis was 1.7 (1.0) years. The mean (SD) baseline CIPN scores were 8.1 (5.6) in the exercise intervention arm and 8.8 (7.9) in the attention control arm (*P* = .56). At 6 months, the self-reported CIPN score was reduced by 1.3 (95% CI, −2.3 to −0.2) points in the exercise intervention arm compared with an increase of 0.4 (95% CI, −0.8 to 1.5) points in the attention control arm. The between-group difference was −1.6 (95% CI, −3.1 to −0.2) points. The point estimate was larger among the 127 patients with CIPN symptoms at enrollment (−2.0; 95% CI, −3.6 to −0.5 points).

**Conclusions and Relevance:**

Findings of this secondary analysis of the WALC trial indicate that a 6-month aerobic exercise intervention vs attention control significantly improved self-reported CIPN among patients who were treated for ovarian cancer. While replication of the findings in other studies is warranted, incorporating referrals to exercise programs into standard oncology care could reduce CIPN symptoms and increase quality of life in patients with ovarian cancer.

**Trial Registration:**

ClinicalTrials.gov Identifier: NCT02107066

## Key Points

**Question:**

What is the effect of exercise on chemotherapy-induced peripheral neuropathy (CIPN)?

**Findings:**

In this secondary analysis of a randomized clinical trial of 134 patients with ovarian cancer, the self-reported CIPN score was 1.6 points lower in those who were randomized to the exercise intervention compared with the attention control group, indicating significant improvement in CIPN symptoms.

**Meaning:**

Findings of this secondary analysis suggest that exercise is a promising treatment for CIPN and incorporating exercise program referrals into the standard oncology care may reduce CIPN symptoms and increase quality of life for survivors of ovarian cancer.

## Teaser

This secondary analysis compared the effect of a 6-month aerobic exercise intervention vs attention control for chemotherapy-induced peripheral neuropathy (CIPN) among participants in the Women’s Activity and Lifestyle Study in Connecticut trial who received chemotherapy for ovarian cancer.

## Introduction

In 2023, there are 19 710 estimated ovarian cancer cases and 13 270 estimated ovarian cancer deaths in the US, making this disease the leading cause of gynecological cancer–related deaths.^1^ Due to the insidious onset of ovarian cancer and the lack of effective screening tools, 60% of patients with ovarian cancer are diagnosed at late stages.^2^ The 5-year survival rates for patients with stage III and stage IV epithelial ovarian cancer are 41% and 20%, respectively.^2^

Approximately 90% of patients with ovarian cancer receive chemotherapy,^3^ and chemotherapy-induced peripheral neuropathy (CIPN) is one of the most common and severe adverse effects of this treatment. The definition of CIPN is damage to sensory, motor, and autonomic neurons^4^ that leads to altered perception of touch, pain, and sense of position and vibration^5^ or to damaged voluntary movement and coordination.^6,7^ Prevalence of CIPN among individuals with ovarian cancer undergoing chemotherapy ranges from 30% to 70%.^8,9,10^ Approximately 60% to 70% of patients receiving paclitaxel, the first-line chemotherapy regimen for ovarian cancer, experience CIPN.^11^ In addition, CIPN can develop or persist after treatment. Approximately 51% of survivors of ovarian cancer reported CIPN symptoms 2 to 12 years after diagnosis.^12^

There is no gold standard for CIPN assessment, and clinical diagnosis is made from patient self-report and medical history.^13^ Because of pain and sensory impairment, CIPN has been associated with worse quality of life^14,15^ and physical function^15^ among individuals with ovarian cancer. Currently, there are no effective drugs or therapies for preventing or treating CIPN,^7,13^ except for duloxetine for painful CIPN, but it has limited benefits.^13^ Approaches for attenuating CIPN during chemotherapy include dose delay, dose reduction, drug substitutions, or stopping chemotherapy.^13^ However, chemotherapy dose reductions and/or delays have been associated with worse ovarian cancer survival.^16,17,18,19,20,21,22,23^ Therefore, pursuit of effective treatment for CIPN is warranted to not only improve quality of life during and after treatment but also potentially increase survival via chemotherapy adherence.

Exercise therapy is a potential treatment for CIPN. A cross-sectional survey found a nonstatistically significant inverse association between physical activity and CIPN in 359 participants treated for ovarian cancer.^24^ Nine small randomized clinical trials (RCTs)^25,26,27,28,29,30,31,32,33^ with no more than 61 participants and 2 larger RCTs with 170 participants^34^ and 350 participants^35^ have investigated the effect of exercise on CIPN symptoms in multiple cancer types both during and after chemotherapy completion, yet CIPN as a composite outcome was comprehensively measured in only 4 of these trials.^26,29,31,34^ Various aspects of CIPN, such as neuropathic pain,^32,33^ balance,^25,28,29,30^ and sensory symptoms,^27,34,35^ were improved in some of the studies. However, a meta-analysis of the 4 trials with CIPN as a composite outcome^26,29,31,34^ found no significant improvement associated with exercise.^36^ To date, no study has examined whether an exercise intervention affects CIPN in patients diagnosed with ovarian cancer. The American Society of Clinical Oncology guideline on CIPN indicated that exercise therapy was potentially beneficial for CIPN treatment, but no recommendation could be made for survivors of any type of cancer due to the lack of evidence.^13^ Thus, research is needed to evaluate the efficacy and clarify the risks of exercise for treating CIPN. In this secondary analysis of the Women’s Activity and Lifestyle Study in Connecticut (WALC) RCT,^37^ we assessed the effect of a 6-month aerobic exercise intervention vs attention control on CIPN among patients who had received chemotherapy for ovarian cancer to provide evidence to inform the guidelines and recommendations for prevention or treatment of CIPN.

## Methods

This prespecified secondary analysis (trial protocol in Supplement 1) was approved by the Connecticut Department of Public Health; the Yale Human Investigation Committees; and the institutional review boards of Dana-Farber/Harvard Cancer Center, Geisinger Health System, and 21 Connecticut hospital sites. All participants provided written informed consent. We followed the Consolidated Standards of Reporting Trials (CONSORT) reporting guideline.

### Recruitment and Randomization

From May 1, 2010, to March 20, 2014, patients who were diagnosed with ovarian cancer and living in Connecticut were recruited to the WALC trial using the Rapid Case Ascertainment Shared Resource of the Yale Cancer Center, a field agent of the Connecticut Tumor Registry that identified patients with ovarian cancer from all hospitals in Connecticut. Individuals were also recruited at 2 additional study sites: Dana-Farber Cancer Institute in Boston, Massachusetts, and Geisinger Health System in Danville, Pennsylvania. Some patients living outside Connecticut learned about WALC trial through national support groups, physicians, or brochures in clinic waiting rooms and were screened via telephone. If eligible and interested, participants completed baseline questionnaires and were randomized 1:1 to a 6-month exercise intervention arm or an attention control arm.

**Figure 1.**
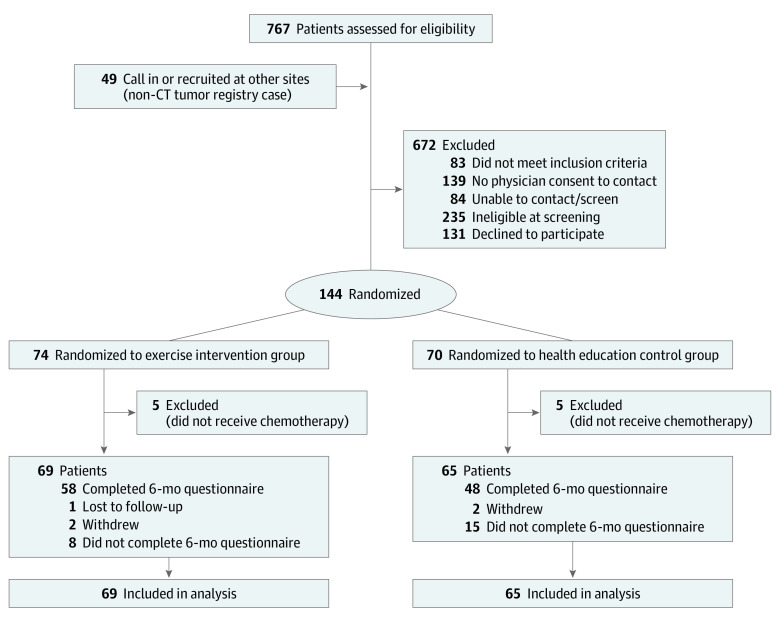
Study Flow Diagram CT indicates Connecticut tumor registry.

Eligibility criteria included (1) ability to speak English, (2) aged 18 to 75 years, (3) diagnosis of stage I to stage IV invasive epithelial ovarian cancer within the past 4 years, (4) completion of chemotherapy (if received) at least 1 month prior to randomization, (5) current exercise routine of less than 90 minutes per week, and (6) receipt of physician consent to start an exercise program. Randomization was block stratified by disease stage (stage I and II vs stage III and IV) and age (≥55 vs <55 years).^37^ Figure 1 shows the study flow diagram.

### Data Collection

Self-reported sociodemographic characteristics, disease stage, time since diagnosis, chemotherapy, treatment status, and history of recurrence were collected at baseline, with additional physician verification of clinical information. Race and ethnicity data were self-reported by participants and included Asian, Hispanic, Native American, non-Hispanic Black, non-Hispanic White, and other (including Hispanic White and non-Hispanic mixed White and African American). Race and ethnicity were collected to ensure the balance of these characteristics between randomized groups. Baseline physical activity levels were assessed using the validated Modifiable Activity Questionnaire.^38^

To determine their CIPN score, participants completed the Functional Assessment of Cancer Therapy/Gynecologic Oncology Group–Neurotoxicity (FACT/GOG-Ntx) scale at baseline and 6 months. The FACT/GOG-Ntx scale provides a measure specifically for CIPN symptoms, including sensory, motor, and auditory problems.^39^ This questionnaire contains 11 items that can be rated on a 5-point Likert scale, from 0 (indicating disagreement: not at all) to 4 (indicating total agreement: very much). Total CIPN scores range from 0 to 44, with higher scores indicating greater CIPN symptoms. The FACT/GOG-Ntx scale has been shown to be reliable, with content validity and concurrent validity and sensitivity to change over time as well as responsiveness to evaluate CIPN among patients treated with chemotherapy.^39^

### Intervention

The exercise intervention consisted of home-based moderate-intensity aerobic exercise facilitated by weekly telephone calls from an American College of Sports Medicine/American Cancer Society–certified cancer exercise trainer. Participants were counseled on increasing exercise to 150 minutes per week of moderate-intensity aerobic exercise, mainly via brisk walking. Adherence was measured using self-reported 7-day daily activity logs on the type and duration of exercise. Participants reported their exercise levels during weekly telephone calls with the exercise trainer. Using a 26-chapter WALC book informed by Social Cognitive Theory,^40^ the exercise trainer provided weekly telephone counseling during which educational topics on exercise and ovarian cancer health were discussed to increase participants’ exercise levels.

The attention control arm received weekly telephone calls from a WALC staff member to discuss ovarian cancer health education topics. Additionally, participants received a 26-chapter book that contained only ovarian cancer survivorship–related information.

### Statistical Analysis

Sample size for WALC was based on detecting a 10% difference in the change in the primary outcome (quality of life).^37^ This sample size achieved 80% power, with α = .05 to detect a standardized effect size of 0.50 (Cohen *d*) between the arms for secondary outcomes, including CIPN.

Baseline characteristics were presented as means (SDs) or percentages and were compared using an unpaired, 2-tailed *t* test, χ^2^ test, or Fisher exact test, as appropriate. A mixed-model repeated measures analysis was conducted to evaluate the 6-month change in CIPN score between participants in the exercise intervention arm vs participants in the attention control arm, and baseline measures were retained as part of the response profile. We chose an unstructured covariance matrix to model the correlations within repeated assessments for the same individual. Linear contrasts were used to obtain the change in CIPN score in each group and group differences, while baseline values of the 2 arms were constrained to be equal.^41^ Least squares means (LS means) and 95% CIs were estimated from the models. Variables associated with missing data at 6 months, including study site and recurrence before and during study, were included as covariates. Results with or without adjustment were similar, thus only adjusted results are presented. The effect of exercise on patients with CIPN symptoms at baseline was examined in a sensitivity analysis. Effect modification by baseline CIPN score, counseling session attendance, and selected baseline characteristics was explored.

All analyses were conducted between September 2022 and January 2023, using SAS, version 9.4 (SAS Institute Inc). Statistical tests were 2-sided, with a *P* < .05 significance level.

## Results

In the WALC trial, 144 participants were randomized to the exercise intervention arm (n = 74) or attention control arm (n = 70). Ten patients (6.9%; 5 in each arm) did not receive chemotherapy, leaving 134 patients for inclusion in this secondary analysis (Figure 1). Among these 134 patients, the mean (SD) age was 57.5 (8.3) years (Table 1). No statistically significant differences in baseline characteristics were observed between the study arms. The mean (SD) time since diagnosis was 1.7 (1.0) years. Most participants had non-Hispanic White race and ethnicity (127 [94.8%]), were employed (67 [50.4%]), were married or living with a partner (99 [73.9%]), and received college or advanced degrees (77 [57.5%]).

**Table 1.** Baseline Characteristics and CIPN Score by Study Arm

Characteristic	Patients, No. (%)^a^		
	Total study population (n = 134)	Study arm	
		Exercise intervention (n = 69)	Attention control (n= 65)
Age, mean (SD), y	57.5 (8.3)	57.2 (9.0)	57.9 (7.8)
Race and ethnicity^b^			
Non-Hispanic White	127 (94.8)	67 (97.1)	60 (92.3)
Other^c^	7 (5.2)	2 (2.9)	5 (7.7)
Educational level			
No high school diploma or GED certificate	4 (3.0)	3 (4.4)	1 (1.5)
High school diploma, GED certificate, and some college or associate’s degree	53 (39.6)	28 (40.6)	25 (38.5)
College or advanced degree	77 (57.5)	38 (55.1)	39 (60.0)
Employment status			
Unemployed or retired	66 (49.6)	32 (46.4)	34 (53.1)
Part-time: <35 h/wk	28 (21.1)	18 (26.1)	10 (15.6)
Full-time: ≥35 h/wk	39 (29.3)	19 (27.5)	20 (31.3)
Marital status			
Single	14 (10.5)	8 (11.6)	6 (9.2)
Divorced, separated, or widowed	21 (15.7)	13 (18.8)	8 (12.3)
Married or living with partner	99 (73.9)	48 (69.6)	51 (78.5)
Live alone	18 (13.4)	9 (13.0)	9 (13.9)
Cancer stage at diagnosis			
Stage I	27 (20.2)	17 (24.6)	10 (15.4)
Stage II	28 (20.9)	10 (14.5)	18 (27.7)
Stage III	57 (42.5)	31 (44.9)	26 (40.0)
Stage IV	21 (15.7)	10 (14.5)	11 (16.9)
Unknown	1 (0.75)	1 (1.5)	0 (0)
Time since diagnosis, mean (SD), y	1.7 (1.0)	1.7 (1.0)	1.7 (1.1)
Cancer recurrence prior to enrollment	23 (17.2)	12 (17.4)	11 (16.9)
Chemotherapy during study^d^	36 (29.0)	16 (25.0)	20 (33.3)
Received carboplatin plus paclitaxel^e^	82 (82.0)	46 (86.8)	36 (76.6)
Study site			
WALC-CT	88 (65.7)	47 (68.1)	41 (63.1)
WALC-G	8 (6.0)	4 (5.8)	4 (6.2)
WALC-DF	7 (5.2)	4 (5.8)	3 (4.6)
WALC-N	31 (23.1)	14 (20.3)	17 (26.2)
Physical activity, mean (SD), min/wk	28.1 (42.4)	25.1 (44.7)	31.2 (39.9)
BMI, mean (SD),	29.4 (7.1)	28.8 (7.0)	30.1 (7.3)
CIPN score, mean (SD)	8.4 (6.8)	8.1 (5.6)	8.8 (7.9)
Neuropathy: CIPN score >0	127 (94.8)	65 (94.2)	62 (95.4)

Abbreviations: BMI, body mass index (calculated as weight in kilograms divided by height in meters squared); CIPN, chemotherapy-induced peripheral neuropathy; GED, General Educational Development; WALC, Women’s Activity and Lifestyle Study in Connecticut; WALC-CT, Connecticut hospitals; WALC-DF, Dana-Farber Cancer Institute; WALC-G, Geisinger Health Systems; WALC-N, National.

^a^ Numbers may not sum to total due to missing data, and percentages may not sum to 100% due to rounding.

^b^ Race and ethnicity data were self-reported by participants.

^c^ The other category comprised 4 Hispanic White, 1 non-Hispanic Black, 1 Hispanic, and 1 non-Hispanic mixed White and African American participants.

^d^ Some participants were receiving maintenance chemotherapy at baseline, whereas some participants went back to chemotherapy due to disease recurrence.

^e^ Overall, 34 participants were missing chemotherapy regimen data.

Most participants were diagnosed at stage III or IV (78 [58.2%]), received carboplatin plus paclitaxel therapy (82 [82.0%]), and did not have disease recurrence (111 [82.8%]). Thirty-six patients (29.0%) received chemotherapy during the study because they were receiving maintenance chemotherapy or went back on chemotherapy due to disease recurrence. The mean (SD) physical activity level at baseline was 28.1 (42.4) minutes per week, and the mean (SD) self-reported body mass index (calculated as weight in kilograms divided by height in meters squared) was 29.4 (7.1). Those who did not receive chemotherapy (n = 10) had similar characteristics to those who received chemotherapy, except for disease stage (eTable 1 in Supplement 2).

Out of a possible total score of 44 on the FACT/GOG-Ntx questionnaire, the mean (SD) CIPN score at baseline was 8.4 (6.8) (exercise: 8.1 [5.6]; control: 8.8 [7.9]; *P* = .56) (Table 1). Additionally, 127 participants (94.8%) had at least 1 CIPN symptom at baseline. Numbness or tingling in feet, discomfort in feet, and joint pain or muscle cramps were the most common CIPN components reported by participants (eTable 2 in Supplement 2).

**Figure 2.**
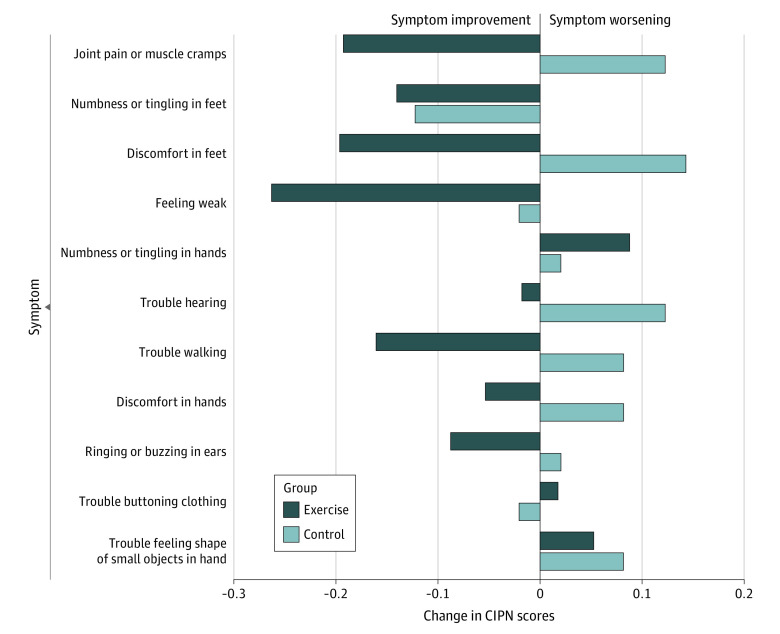
Change in Chemotherapy-Induced Peripheral Neuropathy (CIPN) Component Scores by Study Arm (N = 106)

By the end of the exercise intervention, 58 patients (83.8%) met 80% of the exercise goal, and according to the daily activity logs, the mean (SD) exercise time during the study was 166.0 (66.1) minutes per week. There were no adverse events or reactions reported by participants. After the intervention, the CIPN score was reduced from baseline by 1.3 (95% CI, −2.3 to −0.2) points in the exercise intervention arm, a decrease of 16%. No significant 6-month change in CIPN score was observed in the attention control arm (0.4; 95% CI, −0.8 to 1.5 points) (Table 2). The between-group difference in CIPN score was −1.6 points (95% CI, −3.1 to −0.2 points; *P* = .03). Discomfort in feet, joint pain or muscle cramps, and an overall weak feeling were CIPN components that were reported by participants as being the most improved by the exercise intervention (Figure 2).

**Table 2.** Effect of Exercise Intervention vs Attention Control on CIPN at Baseline and 6 Months

	CIPN score (95% CI)			*P* value (between-group effect)^a^
	Exercise intervention arm (n = 69)	Attention control arm (n = 65)	Between-group difference, LS means (95% CI)^a^	
Among all participants who received chemotherapy (n = 134)				
Common baseline^b^	8.0 (5.6 to 10.4)	8.0 (5.6 to 10.4)	NA	NA
6 mo	6.7 (4.2 to 9.3)	8.4 (5.8 to 11.0)	NA	NA
6-mo Changes	−1.3 (−2.3 to −0.2)	0.4 (−0.8 to 1.5)	−1.6 (−3.1 to −0.2)	.03
Among participants who received chemotherapy and reported CIPN symptoms at baseline (n = 127)				
Common baseline^b^	8.4 (6.0 to 10.8)	8.4 (6.0 to 10.8)	NA	NA
6 mo	6.8 (4.2 to 9.3)	8.8 (6.2 to 11.4)	NA	NA
6-mo Changes	−1.6 (−2.8 to −0.5)	0.4 (−0.8 to 1.5)	−2.0 (−3.6 to −0.5)	.01

Abbreviations: CIPN, chemotherapy-induced peripheral neuropathy; LS means, least squares means; NA, not applicable.

^a^ CIPN scores, exercise effect, and corresponding *P* values were estimated using a mixed-effect model that was adjusted for study site and recurrence before and during study.

^b^ The common baseline levels of CIPN scores were estimated using the mixed-effect model.

In an analysis restricted to patients who reported at least 1 CIPN symptom at baseline (n = 127), a greater CIPN score reduction (−1.6; 95% CI, −2.8 to −0.5 points) was observed in the exercise intervention arm, a decrease of 19%, whereas the change in the attention control arm was similar to that in the primary analysis (0.4; 95% CI, −0.8 to 1.5 points) (Table 2). The overall treatment effect was greater among these 127 participants (−2.0; 95% CI, −3.6 to −0.5 points; *P* = .01). There was no evidence of effect modification by baseline CIPN score, counseling session attendance, disease stage, chemotherapy, baseline body mass index, age, or time since diagnosis (Table 3).

**Table 3.** Exercise Effect on CIPN Stratified by Potential Effect Modifiers (N = 134)^a^

6-mo Change	Study arm participants, No. (%)^b^		CIPN score		
	Exercise intervention (n = 69)	Attention control (n = 65)	Exercise effect, LS means (95% CI)	*P* value	*P* for interaction
Baseline CIPN score^c^					
<7	29 (42.0)	35 (53.8)	−0.9 (−2.9 to 1.2)	.41	.38
≥7	40 (58.0)	30 (46.2)	−2.2 (−4.3 to −0.1)	.05	
Attendance					
≥80%	54 (78.3)	48 (73.8)	−1.7 (−3.3 to −0.1)	.03	.94
<80%	15 (21.7)	17 (26.2)	−1.6 (−5.6 to 2.5)	.44	
Advanced stage (stage III or IV)					
No	27 (39.1)	28 (32.1)	−1.5 (−3.8 to 0.8)	.98	.98
Yes	41 (59.4)	37 (56.9)	−1.5 (−3.6 to 0.5)	.14	
Chemotherapy during study					
No	48 (69.6)	40 (61.5)	−1.6 (−3.3 to 0.2)	.08	.91
Yes	16 (23.2)	20 (30.8)	−1.4 (−4.5 to 1.8)	.39	
Received paclitaxel					
No	11 (15.9)	11 (16.9)	−2.4 (−6.7 to 1.9)	.27	.73
Yes	42 (60.9)	36 (55.4)	−1.6 (−3.5 to 0.3)	.10	
Baseline age, y					
<60	41 (59.4)	38 (58.5)	−2.0 (−4.0 to −0.1)	.04	.51
≥60	28 (40.6)	27 (41.5)	−1.0 (−3.4 to 1.4)	.41	
Baseline BMI: ≥25					
No	24 (34.8)	24 (36.9)	−1.1 (−3.6 to 1.5)	.41	.59
Yes	43 (62.3)	40 (61.5)	−1.9 (−3.8 to −0.0)	.05	
Baseline time since diagnosis, y					
<1	24 (34.8)	23 (35.4)	−2.3 (−4.9 to 0.2)	.07	.51
≥1	45 (65.2)	42 (64.6)	−1.3 (−3.1 to 0.6)	.17	

Abbreviations: BMI, body mass index (calculated as weight in kilograms divided by height in meters squared); CIPN, chemotherapy-induced peripheral neuropathy; LS means, least squares means.

^a^ Values were estimated using a mixed-effect model that was adjusted for study site and recurrence before and during study.

^b^ Numbers may not sum up to 134 due to missing values in potential effect modifiers.

^c^ Median of baseline CIPN score was 7.

## Discussion

We found that a 6-month aerobic exercise intervention significantly improved self-reported CIPN compared with attention control among patients who had completed chemotherapy for ovarian cancer. We observed a significant between-group difference in self-reported CIPN score of −1.6 points, with the score improving by 1.3 points, a reduction of 16%, in the exercise intervention arm; meanwhile, there was a nonsignificant 0.4-point increase among attention control participants. As reported previously, most participants who were randomized to the exercise intervention in the WALC trial were able to meet the exercise goals, with no reported adverse events.^37,42,43^

While data were limited on CIPN specific to survivors of ovarian cancer using the FACT/GOG-Ntx scale, Stevinson et al^24^ reported a mean (SD) CIPN score of 9.9 (0.6) among 192 sedentary patients who had been treated for ovarian cancer, which was slightly higher than the mean (SD) baseline score of 8.4 (6.8) in the present study. The 1.3-point reduction in CIPN score with exercise would correspond with a 1-level improvement on the Likert scale in a specific domain. For example, a 1-level improvement in *discomfort in feet* could indicate that the individual used to feel *quite a bit of discomfort* but now reported *somewhat discomfort*, or a similar change from a little bit to not at all. Previously, we found that aerobic exercise improved health-related quality of life (HRQOL), the primary outcome of the WALC trial, as measured by the Physical Component Summary score of the 36-item Short Form Health Survey, version 1 (RAND Corp).^37^ We assessed the potential relationship between CIPN and HRQOL in this trial population by evaluating the association between change in CIPN score and change in physical or mental HRQOL. We found that improvements in CIPN were distinct from HRQOL improvements as there was no significant association between the change in CIPN and the change in HRQOL.

Four RCTs assessed the effects of exercise on CIPN symptoms in posttreatment survivors of cancer,^30,31,32,33^ but they were limited by smaller sample sizes and shorter study durations compared with the present trial. Kneis et al^31^ compared a 12-week, twice-weekly endurance plus balance training vs endurance training in 50 patients with cancer and persistent CIPN. Four patients were diagnosed with gynecological cancer (cancer types not stated).^31^ No significant group difference was found in CIPN symptoms, which were assessed using objective measurements and self-report, but only 74.0% attained 70% or greater compliance in Kneis et al^31^ compared with 83.8% individuals adhering to 80% of the exercise goal in the present secondary analysis. In Schwenk et al,^30^ the RCT among 22 older individuals (mean age, 70.3 years) with different types of cancer diagnosis (1 with ovarian cancer) and objectively confirmed CIPN compared a 4-week, twice-weekly motor adaptation balance training program with usual care. Sway of hip, ankle, and center of mass were significantly reduced in the intervention group compared with the control group.^30^ Streckmann et al^32^ conducted a 4-arm RCT (with 10 people in each arm) to compare sensorimotor training vs sensorimotor training plus whole-body vibration training vs oncologic control vs healthy control. Among the 30 individuals with cancer, only 3 had ovarian cancer. The CIPN symptoms were objectively measured at enrollment and after 6 weeks of twice-weekly intervention. Tendon reflexes, peripheral deep sensitivity, and pain were significantly improved in the 2 exercise arms compared with the 2 control arms.^32^ Similar to Streckmann et al,^32^ we found improvement in pain, although pain was self-reported in this study whereas CIPN was assessed objectively in both Schwenk et al^30^ and Streckmann et al.^32^ While objective assessment may be important to fully understand CIPN, self-reported symptoms of CIPN best captures patients’ experiences. Dhawan et al^33^ enrolled 45 survivors of cancer with CIPN, 28 of whom had ovarian cancer, to assess the effect of a 10-week home-based muscle strengthening plus balancing exercises vs control on CIPN using self-reports and nerve conduction velocity test. Significant reduction in neuropathic pain scores was observed,^33^ a finding that was consistent with that in the present secondary analysis of the WALC trial.

None of the previous trials^30,31,32,33^ incorporated aerobic exercise. Balance or strength training usually requires certain equipment, but aerobic exercise (such as brisk walking in the present intervention) is more feasible and may be more acceptable for survivors of cancer and requires less cost and effort.

The significant attenuation in CIPN symptoms in the exercise intervention arm could be attributed to both objective improvements in neural functions and subjective improvements in participant perception of these CIPN components. Streckmann et al^32^ reported improved tendon reflexes and peripheral deep sensitivity via objective physical examinations, indicating that exercise might improve neural functions clinically. Physiologically, the potential pathways of aerobic exercise affecting CIPN include increase of blood flow and release of endorphins,^44^ reduction of axonal degeneration, increase of neurotrophic factors, and improvement of mitochondria and oxidative profile.^45^

When patients report CIPN symptoms, chemotherapy dose and schedules may be altered to reduce neurotoxic effects. Since chemotherapy dose reductions and dose delays are associated with higher ovarian cancer mortality,^16,17,18,19,20,21,22,23^ minimizing the dose-limiting effects of CIPN is crucial to potentially improved outcome. Findings of the present secondary analysis suggest that exercise is a viable intervention. However, since the data we used were limited to the posttreatment period, future studies are needed to evaluate the role of exercise in CIPN prevention immediately after cancer diagnosis.

When restricted to participants who had CIPN symptoms at enrollment, we observed a larger effect size with exercise (−2.0; 95% CI, −3.6 to −0.5). Since 94.8% of participants had at least 1 CIPN symptom at baseline, we could not evaluate an interaction by CIPN symptoms at baseline or determine whether the exercise intervention could prevent CIPN among participants without CIPN at enrollment.

### Strengths and Limitations

The WALC RCT, to our knowledge, was the largest randomized study of exercise among patients treated for ovarian cancer and was the only study assessing the effect of exercise on CIPN in this population. We rigorously controlled for the potentially beneficial effect of attention from interventionists with an attention control comparison group. The exercise intervention was mapped directly to the American Cancer Society lifestyle recommendations for survivors of cancer,^46^ and use of self-reported measurement of CIPN corresponded to current clinical practice. Findings indicated the potential for exercise as a minimal risk treatment for CIPN in patients with ovarian cancer after chemotherapy treatment.^13^

This secondary analysis of the WALC trial also had several limitations. More than 90% of the study sample was composed of non-Hispanic White individuals, and all participants spoke English, limiting the ability to generalize findings to diverse populations.^2^ Because CIPN was a secondary outcome for the WALC trial, replication in other ovarian cancer trials with CIPN as the primary outcome is warranted to confirm the benefit we observed. We did not objectively assess CIPN, and a clinically meaningful cutoff point for the self-reported FACT/GOG-Ntx scale has not been described in the literature. Neural function measured by physical examinations would be a valuable addition to better understand the effect of exercise on CIPN. Additionally, we had limited power for subgroup and interaction analyses.

## Conclusions

In the WALC trial, a 6-month aerobic exercise intervention significantly improved self-reported CIPN among patients who had been treated with chemotherapy for ovarian cancer compared with the attention control group. The study provides evidence of the potential benefit of exercise in attenuating a common chemotherapy adverse effect for which there is no accepted treatment. Incorporating referrals to exercise programs into the standard oncology care for patients with ovarian cancer could attenuate CIPN symptoms and increase quality of life. Exercise could also prolong survival by improving chemotherapy adherence, if future studies show the effects of exercise on preventing CIPN during chemotherapy for patients with ovarian cancer.

## Notes

Supplement 1.
Trial Protocol

Supplement 2. **eTable 1.** Baseline Characteristics Among Participants Who Did or Did Not Receive Chemotherapy Prior to Study Enrollment **eTable 2.** Baseline CIPN Component Scores (N=134)

Supplement 3.
Data Sharing Statement
