# Supplementary material for: Effect of Exercise on Chemotherapy-Induced Peripheral Neuropathy Among Patients Treated for Ovarian Cancer: A Secondary Analysis of a Randomized Clinical Trial
Source: JAMA Netw Open. 2023 Aug 1;6(8):e2326463. doi: 10.1001/jamanetworkopen.2023.26463 (PMC10394582; doi:10.1001/jamanetworkopen.2023.26463)
Supplement: Supplement 1. — Trial Protocol [file jamanetwopen-e2326463-s001.pdf]

STUDY PROTOCOL

Title of Research Project: Impact of exercise on ovarian cancer prognosis

RESEARCH PLAN

1. **Statement of Purpose:** State the scientific aim(s) of the study, or the hypotheses to be tested.

Primary Aims: To examine, in 230 women who have completed treatment for Stage I-IV ovarian cancer, the impact of a moderate-intensity aerobic exercise intervention vs. health education on:

- *Quality of Life*
- *Body Composition* (weight, BMI, total and % body fat, waist and hip circumference)
- *Serum Hormones* possibly associated with physical activity and ovarian cancer prognosis (insulin, IGF-I, IGF-II, IGF binding protein-3, CRP, leptin, osteopontin, MIF, prolactin, and CA-125)
- *Diurnal Salivary Cortisol* which is a marker of stress

Secondary Aims:
a) Among the women deemed not eligible or who are not interested in participating in the exercise study, to examine baseline associations among:

- *Quality of Life*
- *Lifestyle (physical activity, diet, weight and BMI)*
- *Demographic and prognostic characteristics (e.g., age, disease stage)*

b) Obtain information on the role of tumor characteristics in the prognosis of ovarian disease in women randomized to the exercise program or the health education program.

c) Validate the lymphedema questionnaire using the volumetric assessment obtained using a Perometer. Evaluate the effect of exercise on lower body lymphedema.

2. **Background:** Describe the background information that led to the plan for this project. Provide references to support the expectation of obtaining useful scientific data.

Ovarian Cancer:
Ovarian cancer is the fourth most frequent cause of cancer death in women, after lung, breast and colorectal cancer (1). It is difficult to treat, as patients frequently present late in the course of the disease, which may be asymptomatic until advanced stages. More than 90% of women with Stage I ovarian cancer will be cured of their disease, but unfortunately only 20% present with localized disease. Advanced disease outside the pelvis portends a poor prognosis and represents the majority of the estimated 15,000 deaths in 2007 (1).

The current standard treatment of ovarian cancer is to optimally debulk the disease surgically and follow with adjuvant chemotherapy. Standard initial therapy most often is five to six courses of systemic chemotherapy with a platinum and taxane regimen. This treatment approach results in a complete clinical response to therapy in 70%-80% of patients with advanced stage disease (31). Despite the often seen dramatic clinical response to treatment, the disease will recur in 60%-85% of patients diagnosed with advanced disease (1). Unfortunately, no proven curative therapy exists for this group of patients, and the optimal treatment approach for those who relapse after initial treatment remains unknown. However, a growing number of new chemotherapeutic agents in recurrent advanced ovarian cancer have been successful at stabilization of disease and thus are allowing recurrent ovarian cancer to be treated as a chronic disease. In turn, the goals of treatment for women with ovarian cancer are to increase survival and disease-free intervals and to improve quality of life.

Late and Long Term Effects of Treatment, Quality of Life (QOL) Issues and Ovarian Cancer:

Because survival time for ovarian cancer has been so short until recently, little opportunity has existed to study or intervene to improve late and long term effects of treatment or QOL among these patients. However, because of advances in therapeutic modalities, now is the opportune time to begin to identify ways that patients maximize their QOL. Studies are needed to develop and test interventions to improve QOL in ovarian cancer survivors.

McCorkle, a co-investigator on the proposed study, and colleagues discussed in their study, "The Silent Killer: Psychological issues in Ovarian Cancer," that because of the stage at presentation as well as aggressive surgery and chemotherapy regimens, vulnerability to psychological morbidity, or poor QOL, is higher in women with ovarian cancer (see Section C.4) (32). Furthermore, research has shown poor QOL, measured shortly after completion of chemotherapy, to be a negative prognostic factor in advanced ovarian cancer for overall survival (33). Significant changes in QOL likely reflect greater disease burden and therefore worse outcome.

QOL for patients with ovarian cancer has also been shown to correlate with the number of physical symptoms, primarily fatigue, experienced (34). The trajectory of fatigue in women with ovarian cancer has been studied; women report that fatigue continues even after completion of treatment (35). Interventions to improve physical symptoms, fatigue and overall QOL in ovarian cancer survivors are paramount. Physical activity has been shown to improve fatigue and other physical symptoms, as well as overall QOL, in breast cancer survivors (8,9,36); yet, no study has examined the effect of increasing physical activity levels on fatigue and QOL in ovarian cancer survivors.

In addition to fatigue, other late and long term effects of ovarian cancer surgery and/or treatment include peripheral neuropathy. The recent landmark report of the results of a large randomized trial demonstrating the impressive favorable impact on survival associated with the administration of a platinum agent plus paclitaxel in recurrent, potentially platinum-sensitive ovarian cancer, has appropriately focused attention on concerns for the development of chemotherapy-induced peripheral neuropathy. In this trial, 20% of patients treated with a platinum agent (generally carboplatin) and paclitaxel developed neuropathic symptoms of grade 2 or greater severity, compared to an incidence of only 1% for women administered single agent platinum therapy. Thus, these data suggest that with the current "standard approach" to the management of treatment-associated neuropathy, women receiving this combination regimen shown to improve survival in recurrent ovarian cancer, have a one in five chance of experiencing discomfort, related to neurological toxicity. The National Cancer Institute (NCI) Common Toxicity Scale defines grade 2 sensory neuropathy to be "objective sensory loss or paresthesia (including tingling), interfering with function, but not interfering with activities of daily living." Once numbness, tingling, and pain develop, the symptoms may persist for months following the discontinuation of treatment, or they may become permanent, even if ultimately reduced somewhat

in severity. Thus, peripheral neuropathy may significantly compromise QOL in ovarian cancer patients. Whether exercise has a favorable impact on QOL, especially among ovarian cancer patients experiencing peripheral neuropathy, is unknown. In the proposed study, we will ask women at baseline and throughout the study if they are experiencing any peripheral neuropathy. If so, then, for women randomized to exercise, we will adapt/modify the exercise program accordingly. For example, we may recommend that the participant exercise with supervision at the health club or cancer survivorship clinics. We may also recommend other activities such as swimming, stationary recumbent bicycling or upper body ergometry.

Lastly, lower body lymphedema has also been reported in some, but not all studies. Most recently, McCorkle (a co-investigator on the proposed study) and colleagues published results from their 6-month randomized clinical trial of a nursing intervention (tailored specialized care to assist patients in developing self-management skills post-ovarian cancer surgery) vs. health education in 145 women diagnosed with ovarian cancer. In their study, none of the women reported lower leg lymphedema at baseline or 6-months. There is a paucity of research on the incidence and impact of lower body lymphedema in the ovarian cancer population. Lymphedema can be an indicator of recurrence and is frequently associated with toxicities such as skin breakdown, pain, neuropathy, and myopathy. Despite these clinical findings, no systematic study of lower body lymphedema in women with ovarian cancer has been conducted. Whether lower body lymphedema is as debilitating and long-term as post-mastectomy lymphedema is not empirically known. For the proposed study, we will adapt or modify the exercise program for women experiencing lower body lymphedema (e.g., if walking at a certain intensity appears to aggravate it, then we will decrease the intensity and/or recommend other activities such as swimming, recumbant bicycling or upper body ergometry (see Section D).

Physical Activity and QOL in cancer survivors:

Several systematic reviews have indicated the importance of physical activity for the physical and psychological health, and overall QOL of cancer survivors (8,9). Randomized trials in breast cancer survivors have suggested that exercise can lead to improvements in QOL, fatigue and cardiovascular fitness. However, few studies have examined physical activity and QOL in ovarian cancer survivors (10). The American Cancer Society has recently recommended that cancer survivors should perform 30 to 60 min of moderate to vigorous physical activity at least 5 days per week (7). The evidence from studies of breast cancer suggests that few cancer survivors reach these targets, but that those that do reach them report higher QOL than inactive survivors (8,9). Recently, Stevinson and colleagues estimated the prevalence of physical activity in ovarian cancer survivors and examined the relationship between physical activity and QOL in 359 ovarian cancer survivors (10). Approximately 31% of ovarian cancer survivors were meeting the public health physical activity guidelines, and being older, having a higher BMI, a diagnosis within the past five years, and a higher stage of disease were associated with lower physical activity levels. Furthermore, women meeting current physical activity guidelines reported significantly better QOL than those not meeting guidelines. Their finding of an approximate 12 point higher QOL associated with higher physical activity levels compared to women with lower physical activity levels is clinically meaningful. A recent chemotherapy trial reported an increase of 10 points in QOL, suggesting that 10 points represents a meaningful improvement in QOL (37). However, due to the observational nature of their study, it is unknown if the strong association between physical activity and QOL indicates that physical activity contributes to QOL; that QOL leads to greater physical activity; or if other variables are responsible for this association. Given the promising results from randomized controlled trials of exercise in breast cancer survivors, their results suggest that a trial examining the potential causal role of physical activity in improving QOL in ovarian cancer survivors is warranted.

Physical activity and ovarian cancer risk and survival:

Physical activity may be associated with not only improved QOL in ovarian cancer survivors, but may also be associated with a lower risk of being diagnosed with ovarian cancer. Recently, Olsen and colleagues systematically reviewed all the available evidence linking physical activity with ovarian cancer (15). Twelve studies were included in the meta-analysis, which gave summary estimates of 0.79 (95% CI, 0.70-0.85) for case-control studies and 0.81 (95% CI, 0.57-1.17) for cohort studies for the risk of ovarian cancer associated with highest versus lowest levels of recreational physical activity. Thus, a modest inverse association exists between level of recreational physical activity and the risk of ovarian cancer. However, no studies have examined the relationship between physical activity after a diagnosis of ovarian cancer and improved disease-free survival. While it remains unclear whether physical activity is associated with prognosis, a growing number of publications, including one of our own, have now shown a strong association between higher levels of physical activity two years after a breast cancer diagnosis and an approximate 50% improvement in disease-free survival (38-40). Breast cancer may share some of the same promotion or growth factors of ovarian cancer, and thus a relationship between higher levels of physical activity and improved ovarian cancer prognosis is quite plausible. While examining the effect of exercise on survival is not a primary aim of the application (because we feel it is important to first examine in a smaller trial the feasibility of participating in exercise after a diagnosis of ovarian cancer, and whether exercise is related to QOL and surrogate markers of survival), if we show that exercise is feasible and associated with improvements in QOL and surrogate markers of survival, then our findings will help guide the design and implementation of a large-scale multi-site trial of exercise on ovarian cancer survival. Such a trial would need to recruit over 1,000 women with ovarian cancer into an exercise program of longer duration (e.g., 3 yrs). However, a strength of our proposed study is that (1) we will have data on whether exercise favorably impacts surrogate markers of survival; (2) we will have preliminary data of the effect of exercise on survival by being able to follow the women, and (3) given we will approach or screen a larger number of ovarian cancer patients than we will randomize, we can also follow these women and examine the longitudinally association between baseline physical activity, BMI, and other variables and survival. In summary, we feel our application is highly significant given it is unknown how well exercise will be accepted in ovarian cancer patients and how much benefit will be obtained in terms of QOL and prognosis. If exercise favorably impacts QOL and prognosis, then exercise may become a routine part of ovarian cancer treatment.

Mechanisms mediating the hypothesized relationship between physical activity and ovarian cancer prognosis:

Several mechanisms have been postulated to explain the hypothesized impact of physical activity upon ovarian cancer risk and prognosis. Some of these include maintenance of body weight/fat and changes in endogenous sex hormones, fasting insulin, IGFs, leptin, C-Reactive Protein (CRP), prolactin, marcrophage inhibitory factor (MIF) and CA-125 (11-14,16-23).

Obesity and Ovarian Cancer Risk and Prognosis:

Overweight has long been recognized as a risk factor for diabetes and cardiovascular disease (41). Most recently, it has become clear that obesity may be the largest avoidable cause of cancer in nonsmokers, accounting for one in five deaths in women in the USA (42). Although many studies have investigated the association between body composition and ovarian cancer risk, results have been inconsistent (43,44). In a pooled analysis of 12 cohort studies, the association between BMI and ovarian cancer risk was examined. The study population consisted of 531,583 women among whom 2,036 epithelial ovarian cancer cases were identified. BMI was not associated with ovarian cancer risk in postmenopausal women, but was positively associated with risk in premenopausal women (43).

This finding is not in agreement with a systematic review from Olsen et al who concluded that overweight and obesity were associated with a 30% increased risk of ovarian cancer (44).

Despite conflicting data regarding ovarian cancer incidence, there are several studies that suggest that obesity is a poor prognostic factor for ovarian cancer survival. One study by Pavelka and collegues identified increased BMI as an independent negative prognostic factor for disease-free survival and overall survival (11), and a second study by Zhang and colleagues reported an association between BMI > 25 kg/m^2 and reduced survival (12). Given that more than 50% of women in the United States are overweight or obese, this finding may have important public health implications. Furthermore, results from a large prospective cohort study in 176,800 postmenopausal women who had never used postmenopausal estrogens showed that overweight and obesity were associated with a 36% increased risk of ovarian cancer mortality (13). Conversely, Barrett and colleagues recently examined the association between BMI and overall survival in 1067 women with ovarian cancer. They found that obese patients with epithelial ovarian cancer do not have a poorer prognosis, provided they receive optimal doses of chemotherapy based on actual body weight (14). However, obese patients with a favorable prognosis are well advised to normalize their weight to avoid the well-known negative general consequences of obesity.

While a number of studies have investigated associations between weight and BMI and ovarian cancer, few have examined weight change on ovarian cancer incidence. Rapp and colleagues conducted a prospective investigation of associations between clinically measured weight change over 5 to 9 years and the incidence of ovarian cancer in a population-based cohort of 36,398 Austrian women. High weight gain was positively associated with ovarian cancer (HR = 2.48; 95% CI: 1.05-5.85) (45).

Possible biological explanations for the observed association between BMI and ovarian cancer mortality involve the potential role of estrogen and androgens in ovarian carcinogenesis (16). Adipose tissue is the primary source of endogenous estrogen after menopause, and circulating levels of estrogen are higher in postmenopausal women who are obese. Both estradiol and estrone stimulate cell growth in normal and malignant ovarian surface epithelial cell cultures. Thus, estrogens from extraglandular sources may promote proliferation and malignant transformation of epithelial ovarian cells. A role of estrogen in the etiology of ovarian cancer is supported by the observed increased risk with long-term use of postmenopausal estrogens and the protective effect of breastfeeding, parity, and oral contraceptive use on ovarian cancer risk (46). Alternatively risk associated with BMI may involve IGFs. Obesity is related to hyperinsulinemia, which increases IGF-I (20).

Insulin, IGFs and Ovarian cancer Risk and Prognosis:

The association of insulin and IGFs to ovarian, breast and other cancers has been established through both basic science and epidemiologic research (18,19,47,48). IGF-1 has potent mitogenic and anti-apoptotic effects that are believed to be involved in the development and progression of ovarian cancer. Two small prospective studies reported a 2- to 5-fold increased ovarian cancer risk among women <55 years old at diagnosis comparing the top versus bottom tertile of IGF-1 levels (47,48). However, Tworoger and colleagues conducted a nested case-control study using data from three prospective cohorts: the Nurses' Health Study (NHS), NHSII, and the Women's Health Study (WHS) to examine whether plasma concentrations of IGF-1, IGFBP-3, and IGFBP-2 were associated with risk of epithelial ovarian cancer (50). Overall, their results did not support a positive association between IGF-related proteins and ovarian cancer risk. Experimental data suggest that IGF-1 may increase ovarian cancer risk by stimulating cell growth and invasive potential; therefore, from a biological perspective, it is unlikely that an inverse association exists between IGF-1 and ovarian cancer risk. Although the IGF system has autocrine/paracrine effects on the ovary, data do suggest that circulating

levels are important in the development of ovarian cancer. Recently, Yu (a co-investigator on the proposed study) and colleagues observed high levels of free IGF-1 peptide to be associated with elevated risk of disease progression in 215 women diagnosed with ovarian cancer (HR = 2.06; 95% CI: 1.22-3.50), and the association was independent of clinicopathologic features of the disease (51).

IGF binding proteins IGFBP-2 and IGFBP-3 may also be important in ovarian cancer etiology. IGFBP-3 is the primary binding protein of IGF-I and levels seem to be lower in women with ovarian cancer versus benign lesions (52). IGFBP-2 is commonly overexpressed by ovarian cancer cells, and retrospective studies report higher IGFBP-2 levels in cases versus controls (53).

IGF-2 may have also be associated with ovarian cancer. To assess the role of IGF-2 in ovarian cancer, Yu and colleagues analyzed IGF-2 expression in ovarian cancer and examined its association with disease characteristics and prognosis in the same cohort of 215 women with ovarian cancer (54). IGF-2 expression was found to be higher in tumors with poor prognosis including tumors with advanced stage, poor differentiation, serous histology and large residual lesions. Women with high IGF-2 had elevated risk for disease progression and death. These findings were also observed in a study by Sayer and colleagues (55). They found expression of the IGF-2 gene to be significantly higher in ovarian cancers relative to normal ovarian epithelium, and that high IGF-2 gene expression was associated with high grade, advanced stage disease, and an independent predictor of poor survival in women with ovarian cancer. While additional research is necessary, these findings suggest that IGF-1 and IGF-2 and their binding proteins may be potential targets for ovarian cancer treatment.

Although studies examining the relationship between insulin levels and ovarian cancer risk and prognosis have not been conducted, several recent studies have demonstrated that elevated fasting insulin levels, which are often seen in obese and inactive individuals (20), are strongly associated with an increased risk of recurrence and death in patients with early stage breast cancer. Goodwin and colleagues demonstrated a two-fold increase in the risk of breast cancer recurrence and a three-fold risk of death in patients with the highest quartile of fasting insulin levels compared to the lowest (56). Two other recent studies, including one of our own (Section C.3.) (57), have demonstrated that women with high levels of c-peptide two years after diagnosis of breast cancer were found to have a lower risk of breast cancer death as compared to women with lower levels of c-peptide (57,58). Breast cancer may share to some extent some of the hormone-related risk factors of ovarian cancer, and thus the etiologic mechanisms of these two diseases may overlap.

Sex Hormones

High insulin and IGF levels are also associated with higher levels of estradiol and lower levels of SHBG (59). The etiology of ovarian cancer appears to be associated with a long-term influence of estrogens (60). Estradiol is produced by the aromatization of androgens within both ovarian follicular granulose and surface epithelial cells, and exerts a significant endogenous role in the development of growing follicles. Several in vivo and in vitro studies suggest that estradiol amy also be involved in the initiation and progression of ovarian cancer (61). Furthermore, protection against ovarian cancer occurs through use of oral contraceptives, multiparity, breast feeding, and hysterectomy, i.e., factors associated with reduced estrogen concentrations (62).

Because endogenous hormones also play a major role in the risk of breast cancer, findings of an association between estrogens and breast cancer risk may also have important implications for ovarian cancer. A pooled analysis of nine cohort studies showed that the risk for breast cancer increased significantly with increasing concentrations of estradiol, free estradiol, and estrone in postmenopausal women (63). A number of clinical trials also show that ovarian ablation increases survival following a diagnosis of breast cancer (64). SHBG levels have also been associated with breast cancer prognosis, especially in premenopausal women (65). SHBG is known to bind to free testosterone and estradiol,

and thus levels of this protein may affect breast and ovarian cancer prognosis through its impact on free hormone levels (26).

Adipocytokines

Recent in vitro data has suggested that several other adipose-related hormones may affect ovarian cancer development and progression. Leptin, a secreted protein of the ob gene by white adipose tissue, plays an important role in energy balance. Leptin has been demonstrated to affect growth of ovarian cancer cell lines, and work is ongoing to determine how these hormones affect ovarian cancer risk and prognosis in vivo (66). A potential role of leptin as an endocrine regulator in ovarian cancer has recently been examined. Choi and colleagues investigated the expression of leptin receptors in immortalized ovarian surface epithelium (IOSE) and ovarian cancer cell lines, and the potential effect of leptin on the cell growth and activation of mitogen-activated protein kinases (MAPKs) in the BG-1 ovarian cancer cell line (67). Both short and long isoforms of leptin receptors were expressed in IOSE-80PC (a post-crisis line), BG-1, OVCAR-3, and SKOV-3 cells. In addition, treatment with leptin resulted in the growth stimulation of BG-1 cells, an activation of ERK1/2 and inhibition of constitutive phosphorylation of p38 MAPK. Their results suggest that further studies are necessary to validate whether leptin may be a potential regulator for ovarian cancer. Lastly, given that leptin is involved in insulin resistance; and can stimulate estrogen biosynthesis by the induction of aromatase activity, from a biological perspective, it is likely that an association exists between leptin and ovarian cancer risk and/or prognosis.

C-Reactive Protein (CRP)

CRP is one of the most important acute-phase proteins produced predominantly by hepatocytes rising rapidly in response to inflammation. CRP has both proinflammatory and anti-inflammatory actions, and it is uncertain which are predominant. Circulating CRP is routinely measured in clinical laboratories as a marker for various acute and chronic inflammatory diseases (68). The pathogenesis and development of ovarian cancer has been closely linked to inflammatory processes. The inflammatory response promotes carcinogenesis by damaging DNA, stimulating angiogenesis and cell proliferation, and inhibiting apoptosis. Ovarian cancer patients with CRP $\leq$ 1 mg/dL vs > 1 have been shown to have an overall 5-year survival of 82% vs. 58.5% ($p < .001$) (69). High serum CRP might reflect a high metastatic potential as inflammation is known to promote metastatic spread by stimulating angiogenesis. CRP has also been shown to be positively correlated with weight and predicts development of type 2 diabetes and cardiovascular disease (70).

Salivary Cortisol

Limited data is available on the diurnal rhythm of cortisol in ovarian cancer patients. However, a large proportion of these patients have been found to have alterations in the diurnal rhythm of cortisol (88), including flattened profiles and high values across the 24-hour cycle. Ovarian cancer patients with altered patterns in cortisol levels have been found to be more likely to have fatigue, poor performance status and vegetative depression (87). In addition to these associations, flattened cortisol patterns have been associated with mortality in metastatic breast cancer.

Other Surrogate/Biological Markers of Ovarian Cancer Risk and Survival

A major strength of our application is the involvement of Dr. Gil Mor, Associate Professor of Obstetrics, Gynecology and Reproductive Sciences at Yale School of Medicine (see Section C). Dr. Mor has been investigating a way to detect ovarian cancer earlier, and he has devised a screening test that measures levels of six cancer-related proteins—leptin, prolactin, osteopontin, IGF-II, macrophage inhibitory factor (MIF), and CA-125—in blood samples. Previous studies had identified each of the six proteins as possible biomarkers of ovarian cancer risk and prognosis. The screen accurately

detected ovarian cancer in 99 percent of cases. The specificity of the test also stood at 99.76 percent (see appendix for reprint). No study has examined the relationship between BMI, physical activity and CA-125, osteopontin, prolactin, and MIF in ovarian cancer patients; nor has a study examined the impact of physical activity on favorably changing these hormones. If we observe a favorable effect of exercise on these surrogate markers of ovarian cancer risk and prognosis, then exercise may become a routine part of the cancer treatment plan.

Effect of physical activity on surrogate markers of ovarian cancer risk and prognosis:

Exercise has been found to lower serum insulin, IGFs, leptin and sex hormone concentrations in postmenopausal women and in breast cancer survivors (17,21, 71). Our research in healthy postmenopausal women, (17,24-28) and our studies in breast cancer survivors, of six months of moderate-intensity aerobic exercise have shown a beneficial effect of physical activity on body fat, insulin, IGFs, sex hormone concentrations, and QOL (see Section C.2) (22,72). While we and others have observed strong associations between low levels of physical activity and obesity with metabolic and sex hormones, no studies have examined these relationships in women with ovarian cancer. With numerous publications showing significant associations between growth factors and sex hormones, and some recent publications showing associations between adipocytokines and inflammation and ovarian cancer risk and prognosis, more effective treatment strategies to reduce hormone levels among ovarian cancer survivors should be explored.

Summary and significance of examining the impact of exercise on QOL and prognosis in ovarian cancer survivors:

The past 20 years have brought undeniable progress in the management of women with ovarian cancer, resulting in improved overall survival. The improved survival rate has increased the importance of improving QOL and maintaining a healthy lifestyle for this population. QOL has received attention in the cancer literature, but a paucity of research involves QOL in long-term ovarian cancer survivors. Regular physical activity has been shown to improve QOL in breast cancer survivors, as well as lower levels of estrogens and increase levels of SHBG. Regular physical activity also significantly lowers insulin levels and enhances insulin sensitivity, and insulin increases the bioactivity of IGF-I. Regular physical activity also helps prevent or reduce obesity with consequent improvement in the metabolic profile. Physical activity may also influence carcinogenesis through alterations in immune system functioning or a reduction in chronic inflammation.

While observational studies have provided an important base of evidence for inferring that physical activity has a protective effect against impaired QOL, these studies cannot, by definition, show a protective “effect” of physical activity against worsening QOL. At this point, clinical trials are needed to (1) determine if exercise at recommended levels is feasible after a diagnosis of ovarian cancer, (2) determine whether the effect of physical activity prevents or attenuates worsening of QOL after a diagnosis of ovarian cancer, and (3) elucidate the biological mechanisms by which physical activity potentially protects ovarian cancer survivors from experiencing recurrence and ovarian cancer-related mortality. To date, no human intervention studies have examined the effect of physical activity on QOL or surrogate markers of prognosis. If randomized controlled exercise trials demonstrate that exercise is feasible and significantly improves QOL and prognosis, exercise could then be prescribed as an integral part of ovarian cancer therapy.

In the observational study we are conducting we will explore the prognostic value of several lifestyle factors. Limited data exists in this area.

3. **Research Plan:** Provide an orderly scientific description of the study design and research procedures as they directly affect the subjects.

D. RESEARCH DESIGN AND METHODS

D.1. Design overview:

A. Parent study

We propose to examine the effect of a randomized controlled exercise intervention vs. health education on QOL and surrogate markers that potentially mediate the relationship between physical activity and ovarian cancer prognosis, in 230 physically inactive women diagnosed with Stage I-IV ovarian cancer. Recruitment will be completed in 3.5 years. Women will be randomized to a home-based exercise program or the health education group. Women randomized to exercise will participate in 150 min/week of moderate-intensity aerobic exercise, such as brisk walking. We will conduct visits at both baseline and six-months plus a 1-year follow up self administered assessment. Compliance with exercise and quality of life will be assessed at the 1-year follow up.

B. Observational study

We will also conduct an observational study, using a one-time mailed questionnaire, with women screened, but deemed not eligible or interested in participating in the exercise trial (approximately 770 women). The observational study will not be conducted at Dana Farber Cancer Institute,at Memorial Sloan Kettering Cancer Institute or at Geisinger Health System facility

D.2. Resources and facilities for performance of the study: The study visits will be conducted at the participant's home and in the study facilities in New Haven. The exercise intervention will take place at home via telephone-based delivery and health clubs around Connecticut (see Section D.13). Hormones will be analyzed at the Yale Center for Clinical Investigation (YCCI).

D.3A. Study Population for Exercise Trial:
Table D.1: Study Inclusion and Exclusion Criteria
Inclusion Criteria:

- Ages 18-75 years at the time of the screening phone call
- AJCC Stages I-IV invasive epithelial ovarian cancer
- Completed adjuvant therapy (i.e. chemotherapy (platinum and a taxane) and/or radiation therapy) at least one month prior to randomization
- Diagnosed within the past four years
- Physically able to exercise and physician consent given to start an exercise program
- Sedentary activity pattern (< 90 mins/week of moderate-to-vigorous intensity recreational physical activity)
- Agrees to be randomly assigned to either of the exercise or the health education group
- Gives informed consent
- Able to travel to New Haven for a baseline and a six-month visit
- Accessible by telephone
- English speaking

Exclusion Criteria:

- Recent (past 6 months) stroke or myocardial infarction

While physical activity may improve QOL several years after diagnosis, we have limited our eligibility to women diagnosed within the past 3 years because of wanting to recruit a somewhat

homogenous sample of women, i.e., women who survive five or more years may differ from women who do not, and the relationship between physical activity, QOL, and prognosis may also differ.

Women must have completed all chemotherapy and radiation at least one month prior to enrollment so that both baseline and follow-up blood analysis are not confounded by whether or not women were receiving chemotherapy.

To observe a maximal effect from the exercise intervention, only women reporting less than 90 min/wk of moderate-to-vigorous intensity sports/recreational physical activity will be eligible for the study. National prevalence data on physical activity indicate that more than three-quarters of American adults report no exercise activity whatsoever. Thus omitting physically active women would not appreciably limit our generalizability. Women with lower average levels of activities, such as in household or occupational settings, even of long durations, would still be eligible. However for all women we will examine eligibility on a per case basis with respect to ability to walk. In summary, our primary exclusion criteria include highly physically active women; thus, we feel our study will be generalizable to most ovarian cancer survivors.

D.3B. Study Population for Observational Study (one-time mailed questionnaire)**:**
Table D.2: Study Inclusion and Exclusion Criteria
<u>Inclusion Criteria:</u>

- AJCC Stages I-IV invasive epithelial ovarian cancer
- Completed adjuvant therapy (i.e. chemotherapy (platinum and a taxane) and/or radiation therapy) at least one month prior.
- Diagnosed within the past three years
- Gives informed consent to complete mailed questionnaire .
- English speaking

D.4A. Recruitment:

Connecticut is a small state geographically, yet has a dense population (~3.2 million), the bulk of which is concentrated in the central portion of the state. Thus, a mechanism was developed for rapidly identifying Connecticut patients with incident cancers. This system, the Rapid Case Ascertainment Shared Resource of the Yale Cancer Center (RCA), has been used by many of our studies. Briefly, RCA staff visit CT's 35 general hospitals on a regular and frequent basis, and search through pathology-department records or hospital tumor-registry records to identify patients with incident cancers. This system allows for the identification of newly-incident cases of cancer more rapidly than they can be reported to the CT Tumor Registry, usually within a few weeks of diagnosis. The RCA activity also facilitates the acquisition of relevant hospital clinical records, pathology slides or tissue blocks for use in studies as appropriate.

In order for RCA to be able to identify all cases of ovarian cancer diagnosed in CT, we must first obtain DPH HIC approval and then obtain IRB approval (or equivalent hospital approval) from each hospital or from DPH HIC after the hospital has indicated that they are not engaged in research.
As of 3/1/2011 final approval to include patients from the following hospitals has been obtained: Bridgeport Hospital, Bristol Hospital, Charlotte Hungerford Hospital, Danbury Hospital, Eastern Connecticut Health Network, Greenwich Hospital, Griffin Hospital, John Dempsey /UCONN (through DPH HIC), Johnson Memorial Hospital, Lawrence and Memorial Hospital, Middlesex Hospital, MidState Medical Center, Hospital of Central Connecticut, New Milford Hospital, Norwalk Hospital, St. Francis Hospital (through DPH HIC), St. Mary's Hospital, Hospital of St. Raphael (through DPH HIC), St. Vincent's Medical Center, Stamford Hospital and William W Backus Hospital.
The DPH HIC is the IRB for St. Francis Hospital and Medical Center, UCONN and the Hospital of St. Raphael.

Annually, about 250 women aged 75 and under, living in CT are diagnosed with epithelial ovarian cancer, of whom ~96% are non-Hispanic White. Women with ovarian cancer will be identified by RCA staff who will visit the pathology department and/or tumor registrar of each of CT's 35 general hospitals approximately every 2-4 weeks. Pathology reports will be obtained for all potentially eligible patients. We will attempt to identify and interview all women meeting the eligibility criteria,

diagnosed with ovarian cancer between the years of 2007 and 2013 (or ~1250 women). A strength of our population-based recruitment approach is that while we are proposing a randomized controlled exercise trial, we also plan to gather data via questionnaire from all women who do not wish to, or are not eligible to participate in the exercise trial. This data will be used to explore the relationship between certain prognostic, demographic, and lifestyle factors. Given approximately 250 women aged 75 and under are diagnosed with ovarian cancer per year, we estimate approaching approximately 1,250 women to randomize 230 women. Following identification of potentially eligible subjects by RCA, her physician will first be contacted via letter to inform him/her that we plan to contact the participant within 2 weeks.

In addition, to patients diagnosed in CT, we will recruit patients diagnosed and/or treated at Dana Farber Cancer Institute, in Boston,Memorial Sloan Kettering Cancer Center in New York and at Geisinger Health System facilities in Pennsylvania. Over 200 newly diagnosed ovarian cancer patients are seen at Dana Farber each year, and over 1000 ovarian cancer patients/survivors are in follow-up or treatment at the Institute. The patient's physician or staff member will describe the study to his/her patient and if the patient is interested in learning more about the study, the physician will obtain consent to release the women's name and contact information to the CT study staff. The patient will then be contacted as described below and screened for eligibility by the CT study staff. (See below for details of the in-person clinic visits at Dana Farber Cancer Institute, Memorial Sloan Kettering Cancer Center and Geisinger Health System facilities).

Finally, we will recruit directly from the community by placing brochures describing the study in the offices of local clinicians.

Study enrollment

We will mail an invitation letter to the participant, describing the study and telling her that one of the study staff will be contacting her within a week to tell her about the study and to solicit her interest and eligibility. If the participant is eligible and interested, a baseline visit will be scheduled either in the subject's home or in the study office. At this point, the physician will also be contacted again via letter to solicit permission for the participant to participate in a monitored, moderate-intensity exercise program. If the participant is currently undergoing chemotherapy, she will be told that she will be called back approximately one month after her chemotherapy has ended. If eligible, women who contact study staff without having received a letter of invitation (women who picked-up a brochure at their clinician's office, or who heard of the study through friends, survivorship support groups, the media etc.) will be randomized following receipt of written permission to exercise from their MD, as with those contacted through the RCA mechanism. To reach more eligible women, we will include information about the study on appropriate internet sites, such as the 'Love/Avon Army of Women recruitment for your study' site.

Our goal is to recruit 230 ovarian cancer survivors over 3.5 years. As mentioned, we will approach approximately 1,250 ovarian cancer survivors for the study to randomize 230 women. Prior pilot studies, performed at Yale, of exercise interventions in cancer survivors, or QOL studies in ovarian cancer survivors have response rates of 15-65%. These rates suggest that our recruitment goals are feasible. Specifically, if we assume 90% survival to recruitment, 95% physician consent, and 75% eligibility, 641 women will be approached for interest of which roughly the first 230 agreeing will be randomized. These rates are based on results from our previous cancer studies, and show that our recruitment is feasible. Even though a subject participation fraction of 35% may be considered on the low side for an epidemiologic investigation, in a randomized controlled trial the first consideration is internal validity, which is generally assured by the randomization. Generalizability could be somewhat less certain, however we will have pathology information and ultimately survival data on essentially all eligible subjects and will be able to compare participants with nonparticipants.

D4.B (Observational study) Among the women from CT screened, but deemed not eligible or interested in participating in the exercise trial, we will invite them to complete a one-time mailed questionnaire

D.5. Recruitment of Women from Ethnic Minority Groups: Given that 96% of women diagnosed with ovarian cancer in Connecticut are non-Hispanic White, we propose to enhance the subject sample for this project to at least 10% African-American and 10% Hispanic. These proportions of women from ethnic minority groups that we will recruit surpass those found in the general population of the study areas. We will ask the oncologists and RCA (when race/ethnicity data is provided) to assist us in recruiting minorities, as this resource does contain race/ethnicity data (we will target minority women early in order to maximize the percent of minority women in the final sample). We will monitor recruitment by creating and reviewing monthly recruitment reports that produce all key recruitment data by race (as well as other variables). We monitor these reports for number of women in each racial group recruited and number of women in each racial group enrolled in the trial. We can shift letters sent, calls initiated, and enrollment offers made based on study experience up to that point. This monitoring will identify the directions for recruitment for the next recruitment effort.

D.6 Data Collection:

<u>D.6.A Parent Study</u> Data collection of study variables will take up to 4.5 years. The total study is 75 years; thus allowing for 6 months lead up time (manual of operations, set-up, recruit, contact physicians, etc). Data collection will involve a screening phone call, a baseline interview most likely conducted in the participant's home (CT) or the Dana Farber Cancer Institute Clinic, the Memorial Sloan Kettering Cancer Center or one of the Geisinger Health System facilities; a baseline and 6-month visit to the study's New Haven, Boston or Geisinger facilities for a DEXA scan, and a 6-month exercise intervention or 6-month health education. A blood sample will be collected at baseline and 6 months. At 1-year women will be mailed a set of self administered questionnaires, which they will complete at home and return in a pre-paid envelope.

D.7. Baseline Data Collection Visits: Baseline data collection will involve a screening phone call, a baseline visit in the participant's home (CT only – in Boston the baseline and clinic visit will be combined), and visit to the study facility in New Haven or Boston for a DEXA scan and blood draw. From past experience we have found that conducting the baseline visit in the participant's home has been most convenient for participants, however this is not practical in the Boston area due to the large distances travelled by some patients Concerns about missed appointments or other issues will be discussed with the participant prior to randomization. If some participants prefer to collapse the two baseline visits then we will do so.

Screening Phone Call: Research staff will call participants within one week after receipt of the letter of invitation and the study brochure. If the participant agrees, the research staff will determine study eligibility. If the participant is eligible, a baseline visit will be scheduled for the following week.

Baseline Visit: Participants will be scheduled for a baseline visit at their home or the study facilities in New Haven or Boston At the visit, research staff will explain the study in detail and then answer any questions. The participant will next sign the informed consent form. The research staff will interview-administer the questionnaires (SEE SECTION D.8 FOR DETAILS) The research staff will instruct the participant on how to complete a 7-Day Physical Activity and Pedometer Log during the next week.

Baseline Clinic Visit : After completion of the baseline visit, in CT the participant will be scheduled (ideally within one week) for a baseline visit at the study center (HRU at YNHH) which will include a DEXA scan to measure total body fat and bone mass and a perometer test to assess leg lymphedema. A binder containing all current IRB approval letters from all participating hospitals will be kept at the

HRU. This can be used by all staff to verify that signed consents from all hospitals are current as some consents are not date stamped.
In Boston the visits will be combined. The participants will also be instructed to fast for 12 hours prior to the visit, not to drink alcohol within 48 hours of the visit, and to refrain from strenuous exercise within 24 hours of the visit. Participants will have ~ 30 ml of blood drawn into 4 vacutainer tubes which will be centrifuged and processed for storage at -70° C as soon as possible following blood draw. Research staff will also review the physical activity and pedometer logs to confirm clarity and completion of the forms. Height, weight, right and left leg circumference and leg length will also be measured.
D.7B Women enrolled in observational study ONLY (CT residents only): Among the women screened, but deemed not eligible or interested in participating in the exercise trial, we will invite them to complete a one-time mailed questionnaire (takes ~20 min to complete). One week after mailing the questionnaire, we will follow up with a phone call to confirm receipt of the questionnaire and to answer any questions regarding the study. A stamped, addressed envelope will be provided.
D.8. Randomization: Women will be randomized into one of 2 study groups using a random permuted block design of varying block size in a 1:1 ratio (N=115 to intervention, 115 to controls). To ensure women with similar characteristics are equivalently assigned to the 2 groups, we will stratify on menopausal status (at diagnosis) and randomize within each stratum. The following study personnel will be masked to participant study group: staff conducting the baseline interview, conducting the DEXA scan and blood draw, reviewing forms and entering data.
D.9. Six-Month Clinic Visit: The same data that were collected at the baseline visits will be collected in a similar manner at the six-month visit. Every effort will be made to have subjects comply. Incentives, such as T-shirts and gift certificates (value of $50), will be given after completion of the six-month clinic visit.
D.9.A 1-Year Follow Up: 1 year following randomization in the study, a set of self administered questionnaires will be mailed to the participant. Questionnaires will include: Quality of Life, Pedometer Log, Physical Activity Questionnaire, Follow Up Questionnaire, and a Daily Activity Log. Participants will return the questionnaires in a pre-paid envelope that will be provided to them.
D.10.A Baseline and Follow-up Measurements (Exercise vs. health education study)
Questionnaires
Information will be collected on demographics, medical history and health habits via a standard questionnaire administered at baseline. At the beginning, during, and upon completion of the study, we will also collect information on diet, exercise preferences, barriers to exercise, and predictors of increased exercise to better inform us for future trials designed to examine the effect of exercise on ovarian cancer prognosis. The Quality of Life questionnaire will be administered at baseline, 3-months and 6-months. We will use questions already developed and used in our previous studies (YES and IMPACT studies; see section C).
Physical Activity: The 7-Day Daily Activity Log (7-Day DAL) will be the primary measure used to compare physical activity between the exercise and control groups. Secondary measures of physical activity will include completion of physical activity questionnaires in all participants. We have extensive experience in measuring physical activity. All physical activity measures we propose to use have been used successfully in our exercise trials in healthy women and breast cancer survivors.

1) Physical Activity Questionnaire: To determine eligibility, at the baseline visit, study participants will be interviewed regarding their past six months of physical activity level. The validated questionnaire used in the YES, IMPACT and HEAL Studies (Section C.2 and C.3) will be used (73). For each activity done, participants will be asked how often and for how long they performed the activity. Hours/week spent in different types (recreational, household, and occupation) and intensities (light, moderate, and vigorous-intensity) of activity will be computed over the past six months. Each activity will be categorized as light (< 3 METs), moderate (3-6 METs), or vigorous (> 6

METs) intensity based on Ainsworth et al's Compendium of Physical Activities (74). We also will examine physical activity levels overall and by intensity between exercisers and the health education participants at baseline and follow-up visits (as a secondary measure of compliance to the study). Change from baseline to follow-up by intervention group will also be determined. While we will only be intervening on moderate- to vigorous-intensity recreational activities, women will report their participation in household, gardening, occupational, and sedentary activities. Thus, we will able to determine if exercise intervention is associated with changes in other types of activity.

2) Seven Day Daily Activity Log (7-Day DAL): The 7-day DAL will also be completed by all participants at baseline (before randomization) and at six-months as our primary measure of study compliance. We will determine hours per week spent in moderate-to vigorous-intensity sports/recreational activities at baseline and follow-up visits, and compare values at baseline and six months between the two study groups. Change from baseline to follow-up by intervention group will also be determined. We also will use data from the DALs as the primary measure of adherence to exercise among women randomized to the exercise group. Exercisers will complete the log daily and turn it in weekly to the exercise trainer for months 1-6 of the trial. This log was used in the Physical Activity for Total Health Study, the Yale Exercise and Survivorship Study, and IMPACT Study (Section C.2) and allowed us to determine weekly adherence over the 6- to 12-months of those randomized exercise trials. The DAL has been shown to measure daily exercise reliably and validly, when compared with physiological measures of compliance to exercise programs, e.g., VO_2 peak (75). When completing the log, women will indicate the sports/recreational activities they performed daily. They also will record the duration of each activity and corresponding heart rate. We will calculate their total minutes per week of moderate- to vigorous-intensity sports/recreational exercise, and then average the weekly minutes over certain time points. We will define good adherence as meeting 80% of the exercise prescription (i.e. 80% of 150 min/week of moderate-to-vigorous intensity sports/recreational exercise). The logs will be discussed during the weekly telephone sessions. We do not plan to use the DALs during the intermediate weeks for women in the study control group, as such usage could provide an inordinate amount of attention to physical activities for participants not in the exercise group.

3) Pedometer: The Yamax pedometer will be used to measure steps walked per day for 7 days at baseline and at six months (as a secondary measure of compliance) in both exercisers and the health education group. The Yamax pedometer has been tested for validity and reliability and has scored high ($r = 0.92$ between pedometer steps/day and VO2max) against other objective and subjective measures of physical activity (76). Participants will be given a form to record the number of steps walked/day. Upon awakening in the morning, participants will attach the pedometer to the belt or waistband and wear it for the entire day (except when bathing or sleeping). Upon retiring to bed at night, participants will take the pedometer off and record steps walked.

4) Quality of Life (QOL): Several QOL questionnaires have been used in ovarian cancer studies. Typically a generic health status validated instrument, such as the FACT-G, is combined with a targeted set of questions designed for patients with ovarian cancer (78). The validated FACT-O questionnaire adds a set of questions for ovarian cancer to the basic FACT-G questionnaire. FACT-COG will also be included. All questions ask about the previous seven days. The physical, functional, and ovarian cancer subscales can be summed to form the Trial Outcome Index (TOI). A total of 39 items are rated on a 5-point Likert scale, with total scores ranging from 0 to 156. Higher scores indicate better QOL. FACT questionnaires are used extensively within the United States and can be administered by self-report or interview (79). For our study, women will be shown how to complete the questionnaire at baseline, but will self-administer it and return it to us at the clinic visit. Research

staff, blinded to randomization group, will review the questionnaire for completeness and clarity. We chose this approach because, based on our previous trials in cancer survivors, women prefer to answer personal questions on their own time in the privacy of their own home.

Fatigue will be assessed using the Fatigue Scale from the FACT questionnaire. Recently Courneya and colleagues (36) showed changes in physical activity were associated with favorable changes in FACT fatigue (p = .002) in their randomized trial of print material and pedometers compared to a control group that was provided physical activity recommendations.

We will also measure QOL with the Short Form Medical Outcomes Survey (SF-36), a comprehensive general survey designed to measure functional health and well-being. It yields an 8-scale health profile which can be summarized into physical and mental health composite scores. The SF-36 has been proven useful in monitoring general and specific populations, comparing the burden of different diseases and differentiating the health benefits produced by different treatments. This questionnaire is used to determine how cancer changes the health profile of patients relative to the general population. In addition, it is a recognized standard tool used in obesity and physical activity research. We will also measure depression at baseline and six-months. Depression will be assessed with the Centers for Epidemiological Studies Depression Scale, a standard measure of depression. This questionnaire had a Chronbach alpha of 0.87 in the YES Study. Happiness will be assessed using the 2-item Fordyce Happiness Measure (HM), which measures the average level of happiness over the past week and the percentage of time an individual has felt happy, unhappy, or neutral over the past week. This measure has been shown to be reliable and valid and to be sensitive to change over time. Self-esteem will be assessed using the Rosenberg Self-Esteem Scale. Depression will measured with the Centers for Epidemiological Studies—Depression Scale (CES-D). Anxiety will be measured using the 20-item State-Trait Anxiety Index (STAI), which differentiates between transient anxiety (‘state anxiety’) and more long-standing anxiety (‘trait anxiety’). Stress was measured using Cohen’s 10-item Perceived Stress Scale a reduction of the original 14-item version that retains good psychometric properties. Uncertainty will be assessed using the Mischel Uncertainty Scale.

5) Theory of Planned Behavior: Prior to randomization, information on intention, attitude, perceived behavior control, and subjective norm will be collected via reliable and valid questionnaires drawn from Ajzen’s Theory of Planned Behavior (80-82) (see Section D.13 for more information on this theory).

6) Dietary Intake: Food frequency questionnaires, assessing the past 6 months, will be administered at baseline and 6-months, as a measure of group dietary patterns. We will use the 120-item FFQ developed and tested in the Women’s Health Initiative multi-ethnic cohort study (83).

7) Lymphedema: Leg lymphedema will be assessed using the Norman Lymphedema Survey, a self-administered questionnaire, at both baseline and follow up visits. Leg circumference will be measured at the baseline and follow up visits to assess changes in lymphedema over the course of the study, a comparison of self-report and the objective measure will be made.

8) Other covariates: Baseline and follow-up values for certain covariates will be determined by a standard questionnaire, to assess for possible confounding of study results. Factors to be included are: reproductive and menstrual history, history of oral contraceptive and hormone replacement therapy use; medication use; history of endocrine problems and other medical problems; family history of specific cancers; history of tobacco; age attained maximum height; lifetime weight patterns and lifetime exercise history.

Anthropometrics: Height, weight, and waist and hip circumference measures will be performed, by research staff blinded to the participant’s randomization group, at baseline and six months. Participants will be weighed in light indoor clothing, without shoes, rounding up to the nearest 0.1 kg; height will

be measured in a standard manner, without shoes, using a stadiometer, rounding up to the nearest 0.1 cm. Circumference measurements will be made at the 1) waist, minimum circumference; 2) umbilicus, and 3) hips (buttock), the greatest circumference. All measures, made by the same staff, will be performed and recorded twice in succession.

Dual Energy X-Ray Absorptiometry (DEXA) scans: DEXA will allow us to measure total fat mass, fat-free mass, and their relative distributions. DEXA scans will be performed at baseline and at 6months. In addition, information on bone mass will be available from the DEXA scan. The DEXA measurements will be made with a Hologic scanner (Hologic QDR 1500, Hologic Inc, Waltham, Mass) that uses a constant potential x-ray source and a k-edge filter (cerium) to achieve a congruent beam of stable dual energy radiation (77). A whole-body scan takes approximately 10 minutes to complete. All DEXA scans will be conducted by a licensed and registered radiologic technologist with specialty certification in Bone Densitometry who will be blinded to the intervention group of the participant. The results will be provided to the participant's physician only upon the participant's request.

Leg volume measurement by Perometer: Women who have had surgery for ovarian cancer are at risk for lower body lymphedema due to lymph node removal. A lymphedema specialist at Yale will conduct a bilateral assessment of leg volume using a Perometer. In addition he/she will do a physical inspection of each limb, the abdomen and feet The measurements will be repeated at 6 months, to evaluate the effect of exercise on lymphedema. The Perometer uses infrared light to scan the leg, which is of minimal risk to the participant. The assessment takes a total of 20 minutes to complete. Women will be asked to remove skirt or pants before the assessment is made.

If the lymphedema specialist identifies level 2 or 3 lymphedema, the participant may be recommended to consult her doctor to determine if she needs a referral to a lymphedema specialist for intervention. Leg lymphedema levels are defined below:

Level 1-no swelling

Level 2-little swelling at end of day, but gone in the am

Level 3-tissue texture changes, swelling does not reduce with elevation

D.10.B Women enrolled in observational study ONLY (CT only)

The one-time mailed questionnaire will contain questions on medical history, demographics, quality of life, physical activity, and diet.

Women enrolled in exercise vs. health education study

Blood draw, Processing, and Storage: Fasting blood (> 12 hours) will be drawn at baseline and six months in a standardized fashion. Two 10ml red-top tubes will be collected for serum, and a 10ml light blue-top and 10ml lavender-top will be collected for citrate and EDTA plasma. Technicians in the lab will centrifuge the samples at 2,000 rpm for 15 minutes at 4°C. Plasma and buffy coat will be separated and transferred into cryovials and labeled with freezer-proof labels with participant ID #s and date. Saliva samples will be collected by each participant for the 4 days prior each clinic visit (baseline and 6 months) and will be stored in the participant's fridge overnight in a sealed plastic bag. Saliva samples will be collected at wakeup and at bedtime. All specimens collected will be stored temporarily at 4°C during transportation or prior to delivery to the site's freezers, and then stored at -70°C until time of analysis. The specimens will be stored in an organized storage system with unique location identifiers based on freezer compartment number, rack number, box number, and slot number. The freezers are configured with beepers, dial-up alarm systems and CO_2 backup systems in the event of a power failure or change in temperature.

Hormones:

Six cancer-related proteins—leptin, prolactin, osteopontin, IGF-II, macrophage inhibitory factor (MIF), and CA-125—will be measured at in the baseline and 6-month blood samples. Previous studies had identified each of the six proteins as possible biomarkers of ovarian cancer risk and prognosis. For the proposed study we will examine the effect of exercise on certain hormones associated with obesity and physical activity in studies of healthy women and women with breast cancer (insulin, IGFs, and sex hormones). We will also examine the effect of exercise on additional biomarkers shown to be associated with ovarian cancer risk and prognosis (i.e., CA-125, osteopontin, prolactin, and MIF).

Baseline and six-month hormones will be measured. Each woman's baseline and follow-up samples will be measured in the same batch, and an equal number of intervention and control samples will also be included in the same batch. Appropriate quality control samples (low and high levels) will be used to monitor the reliability of each assay. Each plate will measure 40 samples and include two sets of quality control (QC) samples. One set of the QC is internal QC that is provided in each kit. Any assay with its internal QC values out of the suggested range will be disregarded and the assay will be repeated. The second set of QC is external QC purchased from a different commercial source. The results of external QC will be analyzed after all measurements are completed. Plates with external QC values greater or less than 2 standard deviation of the mean QC will be repeated. Also, in each assay run, samples with greater than 20% of coefficient of variation in their duplicated results will be repeated. Blind duplicates are also included in and between batches to estimate coefficient of variations.

Insulin: Insulin will be measured via radioimmunoassay using guinea pig antibodies to porcine insulin. ^{125}I-Insulin (porcine) will be used as the radioactive tracer. Samples and serial dilutions of insulin standards (human) will be incubated with antibody and tracer for 4 hours at room temperature. Antibody bound insulin will then be precipitated by a second antibody (goat anti guinea pig gamma globulin), 10% guinea pig serum, and polyethylene glycol and precipitated complex is counted in a gamma counter for measurement and data reduction. The intra- and inter-assay CVs are 6.5% and 9.3%.

IGFs: Plasma concentrations of total IGF-1, IGF-2 and IGFBP-3 will be determined with the use of immunoassays. In order to make our results comparable with the findings published in literature, we will use commercially available immunoassay kits from Diagnostic Systems Laboratories, Inc. (Webster, TX). The analytic methods for total IGFs are enzyme-linked immunosorbent assays (ELISA), which are designed to quantify concentrations of total IGF-1, IGF-2 and IGFBP-3 either in serum or in plasma. Since ELISA employs two specific antibodies to form a sandwich complex with their corresponding antigens, this type of method provides better sensitivity and specificity than other competitive immunoassays. The assays have little cross reactivity with other closely related antigens and the results are reproducible. The assays will be performed following the manufacturer's instructions.

Leptin and CRP: Leptin will be measured by direct RIA using kits from Linco (St. Charles, MO), whereas CRP will be measured with direct chemiluminescent immunoassay on the Immulite analyzer (Diagnostic Products Corporation (DPC, Los Angeles, CA). Intra-/Inter-assay CV percents for each assay range from 4.1 (leptin) to 10.8 (CRP).

CA-125, Osteopontin, Prolactin, and MIF: Given Dr. Gil Mor has performed many assays of these hormones in order to develop his ovarian cancer screening test, he will analyze these specific hormones in his lab. He will use commercially available immunoassay kits from Diagnostic Systems Laboratories, Inc. (Webster, TX). The analytic methods are enzyme-linked immunosorbent assays (ELISA), which are designed to quantify concentrations of these hormones either in serum or in plasma. Since ELISA employs two specific antibodies to form a sandwich complex with their

corresponding antigens, this type of method provides better sensitivity and specificity than other competitive immunoassays. The assays have little cross reactivity with other closely related antigens and the results are reproducible. The assays will be performed following the manufacturer's instructions.

Other Hormone Assays: We also will store blood for future ancillary hormone analyses (e.g., androgens or markers of inflammation). Baseline study consents from participants will allow future ovarian cancer-related analyses with these samples.

Salivary cortisol: Women will be asked to collect 2 saliva samples (wake up and bedtime) for 4 days prior to the baseline clinic visit and the final clinic visit. Analysis of samples will be conducted at YCCI..

Tumor specimens: At baseline, we will request consent from the participants to obtain tumor specimens for future ancillary studies relating to ovarian disease, including investigating the role of tumor characteristics in the prognosis of ovarian disease.

D.11. Medical Record Abstraction and Physician Verification of Treatment Reports: The following will be obtained via a Physician Verification of Treatment Report or abstraction from the medical records: clinical stage, therapy received (chemotherapy/surgery/radiation) and treatment dates.

D.12.A Exercise Intervention: Given the primary side effect of ovarian cancer surgery and treatment is fatigue, and given exercise has had a favorable impact on fatigue in breast cancer survivors, we hypothesize that a similar intervention adapted for ovarian cancer survivors will increase physical activity levels, and this increase in activity will have a favorable impact on fatigue, QOL and surrogate markers of survival. However, given that another symptom reported by ovarian cancer patients may include peripheral neuropathy, we will individualize and modify the exercise program based on any special needs secondary to surgery and treatment (e.g., if the participant is experiencing peripheral neuropathy, then we will recommend supervised exercise at the gym or recommend other activities such as swimming, stationary recumbent bicycling and/or upper body ergometry). The exercise program will also be individualized based on the participant's history of participation in physical activity and her preferred type of exercise.

The exercise intervention will consist primarily of a home-based moderate-intensity aerobic exercise program, e.g., brisk walking. However, our intervention also includes other exercise settings/approaches (e.g., access to health-club, monthly in-person "booster" sessions, monthly physical activity events and newsletters) in an effort to maximize the number of participants who meet and maintain the study's physical activity goals. Because our trial is focusing on efficacy and ensuring a high adherence rate, then it is paramount that we offer a number of different components to increase physical activity participation. If we show a favorable effect of exercise on QOL and prognosis, then future trials may examine effectiveness of exercise and whether one method of increasing physical activity is superior to another.

Exercise Settings/Approaches

Immediately post-randomization: Women randomized to the exercise group will be taught exercise techniques and principles in an initial in-person visit either at their home, at a local gym (we have budgeted for the purchase of a six-month membership for all women randomized to exercise so that participants have daily access to a local gym), or via phone At this visit or phone call, each participant will get to know the exercise trainer, who has the primary responsibility for delivering the intervention (including telephone delivery), eliciting motivation from the participant to achieve goals, and assuring completion of required data collection. The exercise trainer will be a part of the study's personnel, and not a gym employee. While the local gym's exercise trainers will be aware of the trial, we will not be asking them to help with data collection or to carry out the intervention.

Telephone Delivery: Participants will also be taught exercise techniques and behavior change strategies via the telephone (weekly phone calls for months 1-6). The telephone counseling protocol is the principal instrument used to promote exercise adherence in the exercise group. Telephone contact has proven to be effective (and cost effective) for the adoption and maintenance of physical activity in

healthy and cancer populations (65). The study's counseling protocol, based on our previous research (the IMPACT study, Section C.2) and the Cooper Institute's *Active Living Every Day* workbook (based on Project Active) (66), follows a stepwise, phased approach using strategies outlined by social cognitive theory and theory of planned behavior (see section below), indicating that the optimal intervention for a major behavior change should focus on 1) helping the person establish a sequential series of short-term goals and b) enabling the person to review and judge performance on these goals in a way that builds self-efficacy. Our intervention will follow a three-phase protocol: Phase 1 (months 1 and 2) involves education and rapid development of self-efficacy to help participants realize they can change their physical activity; Phase 2 (months 3 and 4) involves self-monitoring and overcoming barriers; and Phase 3 (months 5 and 6) involves motivational reminders and preventing noncompliance. As well as discussions about exercise, the exercise trainer will discuss a health topic of interest each week, information that is given to those in the Health Education group. Inclusion of this information will make the groups comparable with the exception of the exercise intervention.

At the beginning of the study, the participant and exercise trainer will agree upon a regular time to talk on the telephone (see Quality Assurance of Intervention for training of telephone delivery). During each phone call the exercise trainer will discuss the participant's past week physical activity level, any barriers to exercising, and will educate her on how to maintain her exercise program. For the proposed study, the educational material and telephone scripts have already been pilot tested in the IMPACT Study. The participant will record her daily sports/recreational activity in the Daily Activity Log. Every four weeks (after completing the pedometer log), participants will return, via stamped, addressed envelopes, the Daily Activity Log. During the weekly motivational phone calls, the exercise trainer will remind the participant to return her completed Logs to the study office. The physical activity logs will be reviewed by the exercise trainer.

If the participant is not available during the regularly scheduled time to talk, the exercise trainer will make two more phone call attempts later that day or the next day, followed by an email notification (if the participant has access to email). The exercise trainer will document any missed calls and number of attempts to contact the participant. If the exercise trainer is still unable to contact the participant within one week of the scheduled time, then a letter will be mailed to the participant encouraging her to contact the exercise trainer or study staff. Weekly staff meetings between the PI, project manager and exercise trainer will occur, and further discussion as to how to reach the participant will be discussed. Once the exercise trainer contacts the participant, a further discussion of the study goals occurs. Participants are told that meetings, as well as complete, accurate, and timely reports of their physical activity, are critical to the trial's success. Although the study is designed to be primarily a telephone counseling intervention, the exercise trainer will allow flexibility, such that for example, for women who are too busy in a particular week to have a phone call, interaction can be done via email or instant messaging.

Health-Club Delivery: In addition to the telephone counseling, participants will be provided with a six-month gym membership, which will allow them to exercise at a gym especially during inclement weather and in the evenings. The successful Diabetes Prevention Program offered gym access to their lifestyle participants, as does Look AHEAD for participants not adhering to the exercise prescription. The exercise trainer will visit with participants at least monthly either in the gym, the participant's home or neighborhood. Each meeting will feature a theme that promotes adherence and offers novelty while reinforcing the exercise goals prescribed earlier (e.g., "Ways to stay motivated"), in addition to further educating participants on how to exercise safely and at the appropriate intensity.

Voluntary Monthly Events: We also will have voluntary monthly events in CT (e.g., hikes, walks, involvement in local road races) to further motivate the participants to adhere to the exercise program. These events will also provide additional social support and allow the participants to reach particular goals or milestones (e.g., participating in a 5k road race).

Newsletters*:* The quarterly newsletters will feature research updates, physical activity information focused on helping to motivate the participants to maintain the exercise prescription, participant spotlights, and ovarian cancer and general health related information.
Cohort Maintenance Program: Participants will receive other mailings (e.g., birthday cards). In addition, there is the option of "refresher" meetings at the health club or additional individual telephone contact for participants having a difficult time adhering to the exercise prescription.
Exercise Prescription: The exercise intervention will consist primarily of walking with an eventual goal of 150 min/week of moderate-intensity aerobic activity. Our recommended amount of exercise is based on current physical activity guidelines of 30 min/session of moderate-to-vigorous intensity aerobic exercise on most days of the week (84). While we will advise our participants to reach this weekly exercise goal with daily exercise sessions, some women may prefer to achieve this through longer sessions on fewer days. However, we will discourage excessive durations at one session to avoid injuries. All exercise sessions will begin with 5 min stretching warm-up, and will end with 5 min cool down. Exercise will start at 50% of predicted maximal heart rate (220-age) and will be gradually increased in accordance with American College of Sports Medicine guidelines to approximately 60-80% of predicted maximal heart rate (69), with careful tailoring so that older, less fit women will be kept at the lower HR range. Heart rate will be electronically monitored with high/low pulse rate alarms individually set for each subject. Following each exercise session, participants will complete their Daily Activity Log, recording the type of exercise, duration, and average heart rate. The logs will be discussed during the telephone calls, and mailed in monthly to the Study office. If 150 min/week of aerobic exercise was not performed in the previous week, the exercise trainer and participant will discuss barriers experienced by the participant. Participants may participate in strength training, but it will not be counted towards the 150 min/wk goal.
Theory of Planned Behavior: Ajzen's theory of planned behavior is a validated theoretical model that has been used to study exercise behaviors in cancer survivors (85). This theory proposes that intention is the immediate determinant of behavior because it reflects the person's level of motivation and desire to exert effort. In turn, intention is thought to be determined by three independent constructs: attitude, subjective norm, and perceived behavioral control. Perceived behavioral control is defined as the perceived ease or difficulty of performing the behavior, and it may directly predict behavior if it is an accurate reflection of actual control. Attitude is viewed as a positive or negative evaluation of performing the behavior (e.g., good or bad), and subjective norm captures the perceived social pressure that individuals may feel to perform or not perform the behavior. The theory of planned behavior therefore proposes that (a) people will perform a behavior when they intend to do so and have the necessary control over it, and (b) people will intend to perform a behavior when they evaluate it positively, believe that important others think they should perform it, and perceive it to be under their own control. Prior to randomization, participants will complete the Theory of Planned Behavior questionnaire. We will tailor the intervention/telephone counseling based on their responses to the questionnaire and their level of intention. Participants assigned to the exercise group who have weaker intentions will be flagged and given more attention in terms of support and resources for behavior change. This will include highlighting possible incentives for exercise, proactively addressing any anticipated barriers to exercise, and securing social support from family members.
Procedures to Enhance Adherence: 1) Self-monitoring including use of physical activity logs, pedometers, and heart rate monitors delineating intensity, duration, and frequency; 2) Personalized (and written) feedback regarding baseline data with determination of individualized exercise program established from the baseline information; 3) Realistic (individualized) goals that can be updated on a regular basis; 4) Initial use of individual in-person instruction to teach concepts and techniques; 5) Weekly telephone counseling to facilitate overcoming barriers, give positive reinforcement, answer questions and inquire about and assess problems related to exercise; 6) Directly observing participants

exercise during the monthly health club meetings and physical activity events; 7) Group participant support and communication during the monthly meetings/events; 8) Quarterly physical activity summaries of the participant's adherence; and 9) Quarterly newsletters discussing ovarian cancer health and other health topics. Based on our past experience, and the strategies we will use, we expect a high adherence rate among the exercisers (~80%).

Quality Assurance of Intervention

Training and Certification: Dr. Irwin will train the exercise trainer(s) in techniques of telephone counseling and behavior change. Dr. Irwin has experience in conducting exercise trials in healthy women and cancer survivors that resulted in high adherence and compliance to the interventions. The exercise trainers will be required to attend two-day training sessions, conducted by Dr. Irwin, to review implementation of the physical activity protocol and to learn behavior change techniques. Exercise trainers will practice extensive role-playing before conducting their first counseling session. In addition to attending the training sessions, exercise trainers will be instructed to be conversant with the study's protocol and all intervention manuals. Dr. Irwin and/or the project manager will supervise and review at least two individual participant training sessions by each trainer. Exercise trainers will also receive support and training through regular staff meetings, facilitated by the PI and the project manager, to discuss adherence of each participant and to resolve challenging issues.

Summary and Benefits of our Exercise Intervention: Our exercise intervention combines multiple exercise approaches in an effort to maximize the number of participants who meet the study's physical activity goals. The mix of individual and group counseling was selected to reap the potential benefits of each approach. A major strength of the health-club delivery approach is that participants will be able to exercise at times that are convenient to them and in all types of weather. The exercise trainers will also visit the health club at least monthly, allowing them to directly observe the participant exercising. These visits will especially be utilized to assist women who are having difficulty in meeting weekly exercise goals. Individual contact is critical to retaining participants; thus a major strength of using the telephone as the principal instrument of the intervention delivery is personalized and individualized attention and feedback on a regular basis.

D.13.A Attention Control (Health Education) Group: Given one of the primary outcomes is QOL, it is important that the exercise and health education groups are comparable in regards to the amount of attention provided. While, we have designed a control group to be matched on attention and time, it is important that there is no overlap in regards to information on exercise. Furthermore, it is important that the health education group is credible and/or of interest to women diagnosed with ovarian cancer.

We will telephone women randomized to the control group weekly to discuss health issues of interest to these patients. Thus, both exercise and control groups will be matched in regards to attention and time (i.e., weekly phone calls and monthly group meetings), but the content of the interventions will differ (i.e., exercisers will receive all the information the control group receives in addition to exercise training). Furthermore, each group will receive quarterly newsletters, but the content of the newsletters will differ for the exercise and control group. Lastly, incentives ($50 Gift certificate) will be given to participants who comply with the study (i.e., return for 6-month clinic visits), but since these incentives are given at completion of the study, overlap should not be a concern.

Specifically, immediately after randomization, participants in the Health Education Group will be provided written information that emphasizes the importance of a healthy lifestyle. Participants will be encouraged to follow the NCI and ACS physical activity guidelines. This procedure was utilized in our other exercise trials, with no increase in physical activity levels observed at follow-up among women in the health education group. Health Education participants will also receive an equal number of contacts throughout the 6-month intervention as women randomized to exercise. Each week, women randomized to the health education group will receive phone calls to discuss a range of relevant health issues. We will develop handouts that will be discussed at the voluntary monthly meetings. At the end

of the intervention, the exercise trainer will call participants in the health education group to develop a personalized exercise program, which will be mailed to them.

At the end of the study participants in both groups will receive some individual results from the study from the baseline and 6-month visit (BMI, bone mass, body fat, weight, steps per day) and will be given information on survivorship resources in Connecticut.

D.14.Data Processing, Storage, and Confidentiality: We will use a Web-based clinical study data management system that is located on a secure server at Yale University to store and manage study data. Only Dr. Irwin and her staff will have access to the database. Access will be by password protected.

D.15. Study Timeline: Data collection will take 4.5-5 years and will occur from month 6 through month 60. The total study is 5 years; thus allowing for 6 months lead setup time. The timeline we have proposed is similar to timelines followed in our other exercise trials. The first six months will be devoted to preparing questionnaires (note we already have working versions from our previous and ongoing trials), obtaining CT hospital IRB approvals, preparing for recruitment, establishing measurement procedures, planning the exercise intervention, and starting subject identification. Randomization of individuals will start at the beginning of month 6, and will continue until the end of month 48. Data entry and cleaning will be ongoing. Statistical analyses and report writing will begin around month 20 (manuscripts pertaining to study objectives and recruitment). We plan to present preliminary results at conferences in Year 2 - 5 of the study.

D.16. Future Follow Up/Ancillary Studies: We plan to obtain subject consent to recontact them for future studies.

D.16.A Evaluation of Vital Status: The National Death Index will be used to determine vital status of the participants 1-7 years following the completion of the study.

D.17. Strengths and Limitations:

A. Exercise vs health education study - Our study will be the first trial to examine the efficacy of exercise on QOL and prognosis in ovarian cancer patients. Given differences in disease pathology, prognosis, treatment, and side effects of ovarian cancer vs. breast cancer, it may be unwise to simply generalize results observed from exercise trials in breast cancer patients to ovarian cancer patients. Our trial could suggest a unique and important role for exercise in ovarian cancer care given that physical and functional aspects of QOL are often the most compromised in ovarian cancer patients. There is great potential for our findings to make major changes to clinical practice in ovarian cancer patients. Furthermore, while recent observational studies have shown relationships between physical activity and higher QOL in ovarian cancer patients, the temporality of the relationship is unknown. There are many questions regarding whether ovarian cancer survivors will receive clinically meaningful psychosocial and prognostic benefit from exercise. These questions could best be answered by an adequately powered, individually tailored, randomized controlled trial of exercise vs. control on QOL and prognosis. Thus, our proposed study is innovative and very timely. We have excellent experience and preliminary data in recruiting cancer survivors into exercise trials with high adherence and compliance rates and in measuring physical activity, body composition, quality of life (especially in ovarian cancer survivors), hormones and disease-free survival. Our primary eligibility criterion is that women not be currently exercising at recommended levels. Thus, our proposed intervention is likely generalizable to most ovarian cancer survivors. In summary, we believe that the proposed study will provide important scientific and public health information on an exciting area of ovarian cancer survivorship, and is in keeping with the goals of NCI in finding ways to promote physical activity and improve cancer survival and survivorship.

Observational study: All women identified with ovarian cancer throughout CT who do not wish or are not eligible to participate in the exercise study will be invited to participate in this study, making the finding generalizable. The participant burden will be minimal as only a one-time questionnaire.

4. **Statistical Considerations:** Describe the statistical analyses that support the study design.

Statistical Analyses: Exercise vs. health education study. We will perform Analysis of Covariance (ANCOVA), with each woman's change in outcome (final value – baseline) modeled as a function of treatment group. Each variable listed in the Primary Aims section above will be examined in this fashion. The hypotheses will be tested according to the intention-to-treat philosophy in which all randomized participants will be grouped according to their intervention assignment at randomization, regardless of compliance or adherence to the study. Secondarily, mixed effects models for correlated outcomes will also be used to model the data collected on the absolute change in the outcome measures using all time-point differences. Prior to fitting these models, we will perform exploratory data analyses focusing on the distributions of hormone levels by time and intervention group, assessing the appropriateness of log-transformation. Age, prediagnosis menopausal status, race/ethnicity, baseline BMI, and disease stage will be examined as potential covariates to be included in multi-variable adjusted analyses. Among women randomized to exercise, we will also examine effects of exercise on endpoints stratified by adherence (assessed from the DALs) and other measures of compliance (pedometers and PAQ). To explore the potential for effect modification, we will consider stratifying the effect of exercise on endpoints by menopausal status. Two-sided tests will be used for significance. From the food frequency questionnaires, we will identify participants who change their total caloric intake during the study period, and will assess the effect of exercise on outcomes, controlling for change in total caloric intake. Since we are assessing multiple secondary outcomes, we will consider using Bonferroni correction. Although we do not anticipate an appreciable number of participants lost-to-follow-up because of our plan for tracking participants our plan for missing data handling is as follows. The primary mixed model analyses will be performed using all available data, which assumes missing data are missing at random (86). Under this mechanism, the probability of lost-to-follow-up depends on the observed data. Non-random or informative lost-to-follow-up occurs when there is a correlation between the probability of lost-to-follow-up and missing data.

Power and Sample Size Considerations: With a sample size of 230 (which accounts for a potential 30% drop-out/lost to follow up rate) we will have at least 80% power to examine the effect of exercise on QOL and circulating hormones. Our exercise interventions in breast cancer survivors have yielded attrition rates of ~ 10%. Exercise interventions in other cancer survivors (e.g., colon, prostate, and lymphoma) have also yielded similar attrition rates. We hypothesize that attrition may be slightly greater (i.e., 20%) in ovarian cancer patients given their potential for advanced disease, worse QOL and reduced prognosis. However, because no exercise trial has been conducted in ovarian cancer survivors, we now plan to over-recruit to account for a potentially higher attrition rate (30%), and have therefore increased our sample size to N = 230.

In our exercise trial in postmenopausal women, QOL measured by SF-36 was 79.95 $\pm$ 14.88 (72). Assuming a 10% effect size, we would need 54 subjects per group or 108 in total. Taking a 30% potential drop-out rate into consideration, then a sample size of 155 is necessary. A 10% between group effect size was observed in Courneya and colleagues recent publication of physical activity and QOL in ovarian cancer survivors (10). A difference of 10 points is clinically meaningful given a recent chemotherapy trial reported an increase of 10 points in QOL, suggesting that 10 points represents a

meaningful improvement in QOL (37). For insulin, Ligibel recently showed a 28% effect size (2.9 µU/mL) and standard deviation of 8.0 µU/mL, which yields a per group sample size of 80 or total N = 160 (71). Considering the potential for a 30% drop-out with a sample size of 230, a sample size with complete data on 160 women will provide at least 80% power for insulin. For the other hormones, if we assume a 20% effect size, then based on means and standard deviations of these hormones in ovarian cancer survivors (18-20,22,23,47,48,50-55), a sample size of 230 will provide 80% power.

Strategies we will use to achieve a low attrition rate include: 1) Group participant support and communication during the monthly meetings/events; 2) a comprehensive health educational package provided to both groups with weekly topics specifically designed to highlight issues of relevance to ovarian cancer survivors; 3) weekly telephone discussions on topics specific to each participant and their treatment and side effects; 4) quarterly newsletters discussing ovarian cancer health; and 5) incentives. Based on our past experience, and the strategies we will use, we expect a low attrition rate of < 20%.

Observational study. We anticipate 80% of the projected 700 women who are invited to participate in the observational study will do so. Analysis will include logistic regression and survival analysis, investigating predictors of survival.

.

LITERATURE CITED:

1. American Cancer Society. (2006). Cancer facts and figures 2006. Atlanta, GA: American Cancer Society, 2006.
2. Kornblith AB, Thaler HT, Wong G, et al. Quality of life of women with ovarian cancer. Gynecol Oncol 1995; 59: 231-42.
3. Norton TR, Manne SL, Rubin S, et al. Prevalence and predictors of psychological distress among women with ovarian cancer. J Clin Oncol 2004, 22: 919-26.
4. Lockwood-Rayremann S. Survivorship issues in ovarian cancer: a review. Oncol Nurs Forum 2006; 33: 553-62.
5. Guidozzi F. Living with ovarian cancer. Gynecol Oncol 1993; 50: 202-7.
6. Lowe T, Ferrell B, Leong L. Quality of life issues in the management of epithelial ovarian cancer. Curr Treat Options in Oncol 2007; 8: 402-16.
7. Doyle C, Kushi LH, Byers T, et al. Nutrition and physical activity during and after cancer treatment: an American Cancer Society guide for informed choices. CA Cancer J Clin 2006; 56(6): 323-53.
8. Irwin ML, Ainsworth BE. Physical Activity Interventions Following Cancer Diagnosis: Methodologic Challenges to Delivery and Assessment. Cancer Investigation, 2004, 22(1): 30-50.
9. McNeely ML, Campbell KL, Rowe BH et al. Effects of exercise on breast cancer patients and survivors: a systematic review and meta-analysis. CMAJ 2006; 175(1): 34-41.
10. Stevinson C, Faught W, Steed H, et al. Associations between physical activity and quality of life in ovarian cancer survivors. Gynecol Oncol 2007; 106: 244-50.
11. Pavelka JC, Brown RS, Karlan BY, et al. Effect of obesity on survival in epithelial ovarian cancer. Cancer 2006; 197(7): 1520-4.
12. Zhang M, Xie X, Holman CD. Body weight and body mass index and ovarian cancer risk: a case-control study in China. Gynecol Oncol 2005; 98: 228-34.

13. Rodriguez C, Calle E, Fakhrabadi-Shokoohi D, et al. Body mass index, height, and the risk of ovarian cancer mortality in a prospective cohort of postmenopausal women. CEBP 2002; 11: 822-28.
14. Barrett SV, Paul J, Hay A, et al. Does body mass index affect progression-free or overall survival in patients with ovarian cancer? Results from SCOTROC I trial. Annals of Oncology 2008; 19: 898-902.
15. Olsen CM, Bain CJ, Jordan SJ, et al. Recreational physical activity and epithelial ovarian cancer: a case-control study, systematic review, and meta-analysis. CEBP 2007; 16(11): 2321-8.
16. Risch HA. Hormonal etiology of epithelial ovarian cancer, with a hypothesis concerning the role of androgens and progesterone. JNCI 1998; 90: 1774-86.
17. McTiernan A, Tworoger SS, Ulrich CM, et al. Effect of Exercise on Serum Estrogens in Postmenopausal Women: A 12-Month Randomized Clinical Trial. Cancer Research 2004; 64, 2923-28.
18. Khandwala HM< McCutcheon IE< Flyvbjerg A, et al. The effects of insulin-like growth factors on tumorigenesis and neoplastic growth. Endocr Rev 2000; 21: 215-44.
19. Resnicoff M, Ambrose D, Coppola D, et al. Insulin-like growth factor-1 and its receptor mediate the autocrine proliferation of human ovarian carcinoma cell lines. Lab Invest 1993; 69: 756-60.
20. Irwin ML, McTiernan A, Bernstein L, et al. Relationship of obesity and physical activity with c-peptide, leptin, and insulin-like growth factors in breast cancer survivors. Cancer Epidemiology Biomarkers Prevention. 2005; 14(12): 2881-8.
21. Irwin ML, Varma K, Cadmus L, et al. Effect of exercise on fasting insulin and IGFs in breast cancer survivors: The Yale Exercise and Survivorship Study. CEBP, under review.
22. Hefler LA, Concin N, Hofstetter G, et al. Serum C-reactive protein as independent prognostic variable in patients with ovarian cancer. Clin Cancer Res 2008; 14(3): 710-5.
23. Tessitore L, Vizio B, Pesola D, et al. Adipocyte expression and circulating levels of leptin increase in both gynecological and breast cancer patients. Int J Oncol 2004; 24(6): 1529-35.
24. Irwin ML, Yasui Y, Ulrich C, et al. Effect of exercise on total and intra-abdominal body fat in postmenopausal women: A randomized controlled trial. JAMA 2003; 289(3): 323-330.
25. Irwin ML, Tworoger SS, Yasui Y, et al. Influence of demographic, physiologic, and psychosocial variables on adherence to a yearlong moderate-intensity exercise trial in postmenopausal women. Prev Med. 2004 Dec;39(6):1080-6.
26. Frank L, Sorensen B, Yasui Y, Tworoger S, Schwartz R, Ulrich C, Irwin ML, Rudolph R, et al. Effects of Exercise on Metabolic Risk Variables in Overweight Postmenopausal Women. A Randomized Clinical Trial. Obesity Research 2005; 13: 615-25.
27. Irwin ML, Alvarez-Reeves, Cadmus L, et al. Recruiting and retaining breast cancer survivors into a yearlong exercise trial: The Yale Exercise and Survivorship Study 2008, 112(S11):2593-2606.
28. Irwin ML, Cadmus L, Alvarez-Reeves, et al. Effect of exercise on body fat, lean mass, and bone mineral density in breast cancer survivors: The Yale Exercise and Survivorship Study. CEBP, under review.

29. Irwin ML, McTiernan A, Bernstein L, Gilliland G, Baumgartner R, Baumgartner K, Ballard-Barbash R. Physical activity levels among breast cancer survivors. Med Sci Sports Exerc, 2004; 36(9): 1484-1491.
30. Blanchard CM, Courneya KS, Stein K, et al. Cancer survivors' adherence to lifestyle behavior recommendations and associations with health-realted quality of life: results from the American Cancer Society's SCS-II. J Clin Oncol 2008; 26(13): 2198-204.
31. Piccart MJ, Bertelsen K, James K, et al. Randomized intergroup trial of cisplatin-paclitaxel versus cisplatin-cyclophosphamide in women with advanced epithelial ovarian cancer: Three-year results. JNCI 2000; 92: 699-708.
32. McCorkle, R., Pasacreta, J., & Tang. S. T. (2003). Psychological issues in ovarian cancer: An overview and successful nursing intervention. *Holistic Nursing Journal, 17*(6), 1-8.
33. Carey MS, Bacon M, Tu D. The prognostic effects of performance status and quality of life scores on progression-free survival and overall survival in advanced ovarian cancer. Gynecol Oncol 2008; 108: 100-05.
34. Ersek M, Ferrell BR, Dow KH. Quality of life in women with ovarian cancer. West J Nursing Res 1997; 19: 334-50.
35. Payne JK. The trajectory of fatigue in adult patients with breast and ovarian cancer receiving chemotherapy. Oncol Nurs Forum 2002; 29: 1334-40.
36. Mock V, Frangakis C, Davidson N, et al. Exercise manages fatigue during breast cancer treatment: a randomized controlled trial. Psychooncology 2005; 14(6): 464-77.
37. Schink J, Weller E, Harris LS, et al. Outpatient taxol and carboplatin chemotherapy for suboptimaly debulked epithelial carcinoma of the ovary results in improved quality of life: an Eastern Cooperative Oncology Group Phase II Study (E2E93), Cancer J. 2001; 7: 155-64.
38. Holmes MD, Chen WY, Feskanich D, et al. Physical activity and survival after breast cancer diagnosis. JAMA 2005; 293(20): 2479-86.
39. Holick CN, Newcomb PA, Trentham-Dietz A, et al. Physical activity and survival after diagnosis of invasive breast cancer. Cancer Epidemiol Biomarkers Prev. 2008 Feb;17(2):379-86
40. Irwin ML, McTiernan A, Bernstein L, et al. Pre- and post-diagnosis physical activity and mortality in breast cancer survivors: The Health, Eating, Activity, and Lifestyle (HEAL) Study: Journal of Clinical Oncology, In Press.
41. Douketis JD, Sharma AM. Obesity and cardiovascular disease: pathogenic mechanisms and potential benefits of weight reduction. Semin Vasc med. 2005; 5: 25-33.
42. Calle EE, Thun MJ. Obesity and cancer. Oncogene 2004; 23: 6365-78.
43. Schouten LJ, Rivera C, Hunter DJ. Height, body mass index, and ovarian cancer: a pooled analysis of 12 cohort studies. CEBP 2008; 17(4): 902-10.
44. Olsen CM, Green AC, Whiteman DC, et al. Obesity and the risk of epithelial ovarian cancer: a systematic review and meta-analysis. Eur J Cancer 2007; 43: 690-709.
45. Rapp K, Klenk J, Ulmer H. Weight change and cancer risk in a cohort of more than 85,000 adults in Austria. Ann Oncol 2008; 19: 641-48.
46. Hankinson SE, Danforth KN. Ovarian cancer: In Schottenfeld D, Fraumeni JF, editors. Cancer epidemiology and prevention. 3rd ed. Oxford: Oxford University Press; 2006. p. 1013-26.
47. Lukanova A, Lundin E. Toniolo P, et al. Circulating levels of insulin-like growth factor-1 and risk of ovarian cancer. Int J Cancer 002; 101: 549-54.

48. Peeters PH, Lukanova A, Allen N, et al. Serum IGF-1, its major binding protein (IGFBP-3) and epithelial ovarian cancer risk: the European Prospective Investigation into Cancer and Nutrition (EPIC). Endocr relat Cancer 2007; 14: 81-90.
49. blank
50. Tworoger SS, Lee IM, Buring JE, et al. Insuin-like growth factors and ovarian cancer risk: a nested case-control study in three cohorts. CEBP 2007: 16(8): 1691-9.
51. Brokaw J, katsaros D, Wiley A, et al. IGF-1 in epithelial ovarian cancer and its role in disease progression. Growth Factors 2007; 25(5): 346-54.
52. Khandwala HM, McCutcheon IE, Flyvbjerg A, et al. The effects of insulin-like growth factors on tumorgenesis and neoplastic and neoplastic growth. Endocr Rev 2000; 21: 215-44.
53. Lee EJ, Mircean C, Shmulevich I, et al. Insulin-like growth factor binding protein 2 promotes ovarian cancer cell invasion. Mol Cancer 2005; 4: 7-10.
54. Lu L, Katsaros D, Wiley A, et al. The relationship between insulin-like growth factor-2, insulin-like growth factor binding protein-3, and estrogen receptor-alpha expression to disease progression in epithelial ovarian cancer. 2006; 12(4): 1208-14.
55. Sayer RA, Lancaster JM, Pittman J, et al. High insulin-like growth factor-2 gene expression is an independent predictor of poor survival for patients with advanced stage serous epithelial ovarian cancer. Gynecol Oncol 2005; 99(2): 53-4.
56. Goodwin PJ, Ennis M, Pritchard KI, et al. Fasting insulin and outcome in early stage breast cancer: Results of a prospective cohort study. J Clin Oncol 2002; 20: 42-51.
57. Irwin ML, McTiernan A, Bernstein L, et al. Fasting C-peptide levels and breast cancer death: The Health, Eating, Activity and Lifestyle Study. American Association of Cancer Research: Frontiers in Cancer Prevention Meeting, December, 2007.
58. Pollak M, Chapman J, Shepherd L, et al. Insulin resistance, estimated by serum C-peptide level, is associated with reduced event-free survival for postmenopausal women in NCIC CTG MA 14 adjuvant breast cancer trial. J Clin ONcol 24: 9s, 2006 (suppl; abstr 524).
59. Pisani P. Hyper-insulinaemia and cancer, meta-analyses of epidemiological studies. Arch Physiol Biochem. 2008 Feb;114(1):63-70
60. Taube M, Hockenstrom T, Isaksson M, et al. Effect of sex steroids on survival and receptor expression in ovarian cancer epithelial tumor cells. Int J Oncol 2003; 22(6): 1257-62.
61. Chan KK, Wei N, Lui SS, et al. Estrogen receptor subtypes in ovarian cancer: a clinical correlation. Obstet Gynecol 2008; 11(1): 144-51.
62. Risch HA. Hormonal etiology of epithelial ovarian cancer, with a hypothesis concerning the role of androgens and progesterone. JNCI 1998; 90: 1774-86.
63. Endogenous Hormones and Breast Cancer Collaborative Group. Endogenous sex hormones and breast cancer in postmenopausal women: reanalysis of nine prospective studies. J Natl Cancer Inst 2002; 94:606-616.
64. Adjuvant breast cancer trials collaborative group. Ovarian ablation or suppression in premenopausal early breast cancer: results from the international adjuvant breast cancer ovarian ablation or suppression randomized trial. J Natl Cancer Inst. 2007 Apr 4;99(7):516-25.
65. Clemons M, Goss P. Estrogen and the risk of breast cancer. N Engl J Med 344(4):276-85, 2001 (review).

66. Tessitore L, Vizio B, Pesola D, et al. Adipocyte expression and circulating levels of leptin increase in both gynecologic and breast cancer patients. Int J Oncol 2004; 24(6): 1529-35.
67. Choi JH, Park SH, Leung PC, et al. Expression of leptin receptors and potential effects of leptin on the cell growth and activation of mitogen-activated protein kinases in ovarian cancer cells. J Clin Endocrinol Metabl 2005; 90(1): 207-10.
68. Marnell L, Mold C, Du Clos TW, et al. C-reactive protein: ligands, receptors and role in inflammation. Clin Immunol 2005; 117: 104-11.
69. Hefler LA, Concin N, Hofstetter G, et al. Serum c-reactive protein as independent prognostic variable in patients with ovarian cancer. Clin Cancer Res 2008; 14(3): 710-7.
70. Deodhar SD. C-reactive protein: the best laboratory indicator available for monitoring disease activity. Cleve Clin J Med. 19898: 56: 126-30.
71. Ligibel JA, Campbell N, Partridge A, et al. Impact of a mixed strength and endurance exercise intervention on insulin levels in breast cancer survivors. J Clin Oncol. 2008 Feb 20;26(6):907-12
72. Bowen DJ, Fesinmeyer MD, Yasui Y, et al. Randomized trial of exercise in sedentary middle aged women: effects on quality of life. Intern J Beh Nutr PA. 2006; 3 (34): 5678-84.
73. Kriska A. Modifiable activity questionnaire. Med Sci Sports Exer 29; S73-78: 1997.
74. Ainsworth BE, Haskell WL, Whitt MC, Irwin ML, Swartz AM, Strath SJ, O'Brien WL, Bassett DR, Schmitz KH, Emplaincourt PO, Jacobs DR, Leon AS. Compendium of physical activities: An update of activity codes and MET intensities. Medicine and Science in Sports and Exercise 2000; 32(9): S498-S516.
75. Blair S, Haskell W, Ho P, et al. Assessment of habitual physical activity by a Seven-Day Recall in a community survey and controlled experiments. Am J Epidemiol 1985; 122: 794-804.
76. Bassett D, Ainsworth B, Leggett S, et al. Accuracy of five electronic pedometers for measuring distance walked. Med Sci Sport Exer 1996; 28: 1071-7.
77. Pietrobelli A, Formica C, Wang Z, and Heymsfield S. Dual-energy X-ray absorptiometry for total-body and regional bone-mineral and soft-tissue composition. Am J Physiol. 1996; 271: E941-951
78. Cella DF, Tulsky DS, Gray G, et al. The functional assessment of cancer therapy scale: development and validation of the general measure. JCO 1993; 39: 939-50.
79. Fallowfield LJ, Leaity SK, Howell A, et al. Assessment of quality of life in women undergoing hormonal therapy for breast cancer: validation of an endocrine symptom subscale for the FACT-B. Breast C Res Treat 1999; 55: 189-99.
80. Azjen I. The theory of planned behavior. Organ Behav Hum Decis Processes. 1991; 50: 179-211.
81. Hagger MS et al. A meta-analytic review of theories of reasoned action and planned behavior in physical activity: predictive validity and the contribution of additional variables. Exercise Psych, 2002; 24: 3-32.
82. Courneya, K.S., Karvinen, K.H., & Vallance, J.K.H. (in press). Exercise motivation and behavior change. In M. Feuerstein (Ed.), Handbook of Cancer Survivorship (pp. xxx-xxx). New York, NY: Springer.
83. Patterson Re, Kristal A, Tinker L, et al. Measurement characteristics of the Women's Health Initiative food frequency questionnaire. Ann Epidemiol 1999; 9(3): 178-87.

84. Pate R. Guidelines for Exercise Testing and Prescription, 4th Edition. Philadelphia: Lea & Febiger 1991. p61.
85. Courneya K, Friedenreich C. Utility of the theory of planned behavior for understanding exercise during breast cancer treatment. Psycho-Oncology 1999; 8: 112-22.
86. Rubin DB. Inference and missing data. Biometrika 1976; 63:581-590.

87. Weinrib AZ, Sephton SE, DeGeest K et al. Diurnal Cortisol Dysregulation, Funcitonal Disability, and Depression in Women with Ovarian Cancer. Cancer 2010; 116: 4410-4419

88.Touitou Y, Bogdan A, Levi F, et al. disruption of the circadian patterns of serum cortical in breast and ovarian cancer patients; relationships with tumour marker antigens. J Cancer Res Clin 1995; 121;181-188.
