# Supplementary material for: Effect of Exercise on Chemotherapy-Induced Peripheral Neuropathy Among Patients Treated for Ovarian Cancer: A Secondary Analysis of a Randomized Clinical Trial
Source: JAMA Netw Open. 2023 Aug 1;6(8):e2326463. doi: 10.1001/jamanetworkopen.2023.26463 (PMC10394582; doi:10.1001/jamanetworkopen.2023.26463)
Supplement: Supplement 2. — eTable 1. Baseline Characteristics Among Participants Who Did or Did Not Receive Chemotherapy Prior to Study Enrollment eTable 2. Baseline CIPN Component Scores (N=134) [file jamanetwopen-e2326463-s002.pdf]

Supplemental Online Content

Cao A, Cartmel B, Li FY, et al. Effect of exercise on chemotherapy-induced peripheral neuropathy among patients treated for ovarian cancer: a secondary analysis of a randomized clinical trial. *JAMA Netw Open*. 2023;6(8):e2326463. doi:10.1001/jamanetworkopen.2023.26463

eTable 1. Baseline Characteristics Among Participants Who Did or Did Not Receive Chemotherapy Prior to Study Enrollment

eTable 2. Baseline CIPN Component Scores (N=134)

This supplemental material has been provided by the authors to give readers additional information about their work.

© 2023 Cao A et al. *JAMA Network Open.*

eTable 1. Baseline Characteristics Among Participants Who Did or Did Not Receive Chemotherapy Prior to Study Enrollment[a]

Characteristics	Received chemotherapy (N=134)[b]	Did not receive chemotherapy (N=10)[b]	P [c]
**Age**	57.5±8.4	55.1±11.6	0.40
**Race/ethnicity**			0.60
Non-Hispanic white	127 (94.8)	10 (100.0)	
Other	7 (5.2)	0 (0.0)	
**Education Level**			0.17
No GED or Equivalent	4 (3.0)	0 (0.0)	
GED and Some College/Associates	53 (39.6)	7 (70.0)	
College Graduate or Advanced Degree	77 (57.5)	3 (30.0)	
**Employment Status**			0.18
Unemployed/Retired	66 (49.6)	3 (30.0)	
Employed Part Time (< 35 hrs/wk)	28 (21.1)	1 (10.0)	
Employed Full Time (>= 35 hrs/wk)	39 (29.3)	6 (60.0)	
**Marital Status**			0.48
Single	14 (10.5)	1 (10.0)	
Divorced, Separated, or Widowed	21 (15.7)	3 (30.0)	
Married or Living with Partner	99 (73.9)	6 (60.0)	
**Cancer Stage at Diagnosis**			**<0.001**
Stage I	27 (20.2)	7 (70.0)	
Stage II	28 (20.9)	2 (20.0)	
Stage III	57 (42.5)	1 (10.0)	
Stage IV	21 (15.7)	0 (0.0)	
Unknown	1 (0.8)	0 (0.0)	
**Time Since Diagnosis (years)**	1.7±1.0	1.6±0.9	0.59
**Chemotherapy during Study**	35 (26.1)	0 (0.0)	0.14
**Cancer Recurrence Prior to Enrollment**	23 (17.2)	2 (20.0)	0.68
**Live Alone**	18 (13.4)	1 (10.0)	1.00
**Study Site**			0.91
Yale	88 (65.7)	7 (70.0)	
Geisinger	8 (6.0)	0 (0.0)	
Dana-Farber	7 (5.2)	0 (0.0)	
National	31 (23.1)	3 (30.0)	
**Physical Activity (min/wk)**	28.1±42.4	32.1±30.8	0.77

Abbreviations: CIPN: Chemotherapy-induced peripheral neuropathy; DFCI: Dana-Farber Cancer Institute.

[a] Table values are mean ± SD for continuous variables and n (column %) for categorical variables.

[b] Numbers may not sum to total due to missing data, and percentages may not sum to 100% due to rounding.

[c] P-value is for t-test (continuous variables), $\chi 2$ test (categorical variables), or Fisher's exact test (cell counts <5).

© 2023 Cao A et al. *JAMA Network Open.*

eTable 2. Baseline CIPN Component Scores (N=134)

Question Number	Question Description	Self-reported Scores[a]					Average[b]
		0	1	2	3	4	
**1**	Numbness or tingling in hands	78 (58.2)	34 (25.4)	13 (9.7)	7 (5.2)	2 (1.5)	0.7 ± 1.0
**2**	Numbness or tingling in feet	48 (35.8)	30 (22.4)	28 (20.9)	21 (15.7)	7 (5.2)	1.3 ± 1.3
**3**	Discomfort in hands	85 (63.4)	24 (17.9)	7 (5.2)	15 (11.2)	3 (2.2)	0.7 ± 1.1
**4**	Discomfort in feet	52 (38.8)	33 (24.6)	21 (15.7)	20 (14.9)	8 (6.0)	1.3 ± 1.3
**5**	Joint pain or muscle cramps	42 (31.3)	32 (23.9)	27 (20.2)	19 (14.2)	14 (10.5)	1.5 ± 1.3
**6**	Feel weak	73 (54.5)	40 (29.9)	15 (11.2)	5 (3.7)	1 (0.8)	0.7 ± 0.9
**7**	Trouble in hearing	83 (62.4)	26 (19.6)	11 (8.3)	7 (5.3)	6 (4.5)	0.7 ± 1.1
**8**	Ringing or buzzing in ears	90 (67.2)	18 (13.4)	12 (9.0)	6 (4.5)	8 (6.0)	0.7 ± 1.2
**9**	Trouble in buttoning buttons	113 (84.3)	13 (9.7)	4 (3.0)	2 (1.5)	2 (1.5)	0.3 ± 0.7
**10**	Trouble in feeling the shape of small objects in hand	121 (90.3)	7 (5.2)	3 (2.2)	2 (1.5)	1 (0.8)	0.2 ± 0.6
**11**	Trouble in walking	85 (63.4)	34 (25.4)	10 (7.5)	4 (3.0)	1 (0.8)	0.5 ± 0.8
**Total**	Overall CIPN	-	-	-	-	-	8.4 ± 6.8

Abbreviations: CIPN: Chemotherapy-induced peripheral neuropathy
[a] N (row %) for each question
[b] Mean ± standard deviation

© 2023 Cao A et al. *JAMA Network Open.*
