# Supplementary material for: Effect of Exercise on Chemotherapy-Induced Peripheral Neuropathy Among Patients Treated for Ovarian Cancer: A Secondary Analysis of a Randomized Clinical Trial
Source: JAMA Netw Open. 2023 Aug 1;6(8):e2326463. doi: 10.1001/jamanetworkopen.2023.26463 (PMC10394582; doi:10.1001/jamanetworkopen.2023.26463)
Supplement: Supplement 3. — Data Sharing Statement [file jamanetwopen-e2326463-s003.pdf]

Data Sharing Statement

Cao. Effect of Exercise on Chemotherapy-Induced Peripheral Neuropathy Among Patients Treated for Ovarian Cancer. *JAMA Netw Open*. Published August 01, 2023.
doi:10.1001/jamanetworkopen.2023.26463

Data
Data available: Yes
Data types: Other (please specify)
Additional Information: The datasets generated and/or analyzed during the current study are available from the corresponding author on reasonable request.
How to access data: The datasets generated and/or analyzed during the current study are available from the corresponding author on reasonable request.
When available: With publication

Supporting Documents
Document types: None

Additional Information
Who can access the data: Researchers whose proposed use of the data has been approved by the WALC research team.
Types of analyses: Any purpose that has been approved by the WALC research team.
Mechanisms of data availability: With investigator support.
